# The functional aspects of selective exposure for collective decision-making under social influence

**DOI:** 10.1038/s41598-024-56868-8

**Published:** 2024-03-17

**Authors:** Poong Oh, Jia Wang Peh, Andrew Schauf

**Affiliations:** 1 Wee Kim Wee School of Communication and Information, Nanyang Technological University, Singapore, 639798 Singapore; 2 Information Systems Technology and Design Pillar, Singapore University of Technology and Design, Singapore, 487372 Singapore; 3 NUS Cities, College of Design and Engineering, National University of Singapore, Singapore, 117566 Singapore

**Keywords:** Human behaviour, Dynamic networks, Modularity, Numerical simulations

## Abstract

Opinion diversity is crucial for collective decision-making, but maintaining it becomes challenging in the face of social influence. We propose selective exposure as an endogenous mechanism that preserves opinion diversity by forming exclusive subgroups of like-minded individuals, or echo chambers, which have been often perceived as an obstacle to achieving collective intelligence. We consider situations where a group of agents collectively make decisions about the true state of nature with the assumption that agents *update* their opinions by adopting the aggregated opinions of their information sources (i.e., naïve learning), or alternatively, *replace* incongruent sources with more like-minded others without adjusting their opinions (i.e., selective exposure). Individual opinions at steady states reached under these dynamics are then aggregated to form collective decisions, and their quality is assessed. The results suggest that the diversity-reducing effects of social influence are effectively confined within subgroups formed by selective exposure. More importantly, strong propensities for selective exposure maintain the quality of collective decisions at a level as high as that achieved in the absence of social influence. In contrast, naïve learning allows groups to reach consensuses, which are more accurate than initial individual opinions, but significantly undermines the quality of collective decisions.

## Introduction

The independence of individual opinions is crucial for collective decision-making because it ensures opinion diversity within groups^1–5^. However, maintaining opinion diversity is challenging because social influence among group members tends to reduce it^6–8^. Empirical studies have suggested the diversity-reducing effects of social influence in various settings since Asch’s seminal study on social conformity, showing that groups under social influence tend to arrive at biased collective decisions^9–14^. Nevertheless, it has been documented that real-world groups perform well in decision-making across a wide range of domains, for example, from crowd voting for product designs^15^ and stock price prediction^16^ to COVID-19 detection^17^ more recently. It is important to note that all these successful collective decisions were made in highly interactive communication environments where social influence was not just possible but facilitated, and where, therefore, the independence of opinions was hardly expected. The question then becomes, “How can groups perform well in decision-making *despite* social influence?” To address this question, we propose *selective exposure* as a plausible endogenous mechanism that can effectively manage the diversity-reducing effects of social influence to preserve opinion diversity at the global level, thereby maintaining the quality of collective decisions.

We consider situations where a group of agents collectively make decisions about the true state of nature by utilizing information distributed in the form of opinions held by individual members. Assuming that the true state remains *unknown* to individual agents, we exclude from our model self-correction, incentives, and payoffs based on the accuracy of opinions, whereas these factors are considered in previous studies^6,8,18–22^. Further, we assume that agents’ initial opinions are *independent* and distributed around the true state with non-trivial *errors*. The errors in initial opinions, however, cancel each other out when they are aggregated, for example, by the majority rule^23^, median aggregation^24^, or mean aggregation^25^. In our model, therefore, a simple aggregation of initial opinions held by group members can yield sufficiently accurate collective decisions, with the collective accuracy increasing as group size grows, thereby achieving “collective intelligence”. The accuracy achieved by a simple aggregation of initial opinions is considered the *null model* (i.e., the quality of collective decisions that would have been achieved *without* social influence), against which we assess the qualities of both individual and collective decisions formed *under* social influence.

We formulate *social influence* following DeGroot’s model of opinion dynamics^26^, where agents naïvely update their opinions by repeatedly adopting the majority or average opinion of their information sources (i.e., naïve learning). In some previous studies, naïve learning is considered as a means for agents to minimize the discrepancies with their information sources (i.e., normative social influence)^27,28^. At the same time, naïve learning can also be seen as a useful heuristic for agents to improve their opinions when access to information is limited. This is because an opinion updated through naïve learning is a “collective” decision in the sense that it is made by aggregating the opinions in one’s information pool at a small and local scale (i.e., informational social influence)^29,30^. However, the repetition of naïve learning necessarily reduces opinion diversity within groups over time, regardless of whether it exerts normative or informational social influence, illustrating the diversity-reducing effects of social influence. In each round, the agent with the most extreme opinion is exposed to less extreme opinions than its own, and its opinion becomes less extreme through naïve learning in the following round. Especially when the patterns of influence from information sources to recipients are represented by a directed network, and the network is strongly connected (i.e., every agent is connected to everyone else by at least one directed path), all agents end up adopting a common opinion, and so the group reaches a consensus, as DeGroot’s theorem states. We consider the convergence of opinions at steady states reached through naïve learning as the state where there is a lack of opinion diversity within groups.

A group consensus reached through naïve learning is generally not equal to the collective decision formed by a simple aggregation of initial opinions in the group^31^. Instead, it is determined by the *weighted* mean of initial opinions, where the weights are proportional to the eigenvector centralities of corresponding agents in the network^32^ and represent the agents’ relative influences on the consensus. Golub and Jackson^33^ and Acemoglu et al.^34^ show that naïve learning can still produce accurate group consensuses in the absence of “excessively” influential agents, modeled as hubs in wheel-shaped networks. However, the degree distributions and the distributions of eigenvector centralities of real-world social networks typically follow power laws^35^. Further, for social networks that are constantly growing, central nodes tend to become more central through preferential attachment processes^36,37^ in contrast to the assumption made by Acemoglu et al.^34^ Therefore, a well-connected large group is expected to reach a consensus that significantly deviates from the simple aggregation of initial opinions. In addition, those studies (also Buechel et al.^30^ and Rauhut and Lorenz^38^) adopt rather “generous” assessment criteria (i.e., the deviation of group consensuses from the true state) without considering that the marginal improvement of collective accuracy necessarily decreases as group size increases^39^. When the effects of group size on collective accuracy are not accounted for, the effects of influential agents in large groups tend to be underestimated.

Other studies focus on individual attributes of agents, specifically stubbornness, as another potential source of the deviation of group consensuses from the true state. For instance, Acemoglu et al.^34^ show that the presence of “forceful” agents—those who influence others but do not change their own opinions—results in a consensus biased toward the initial opinions of the forceful agents, increasing the deviation. Similarly, Buechel et al.^30^ suggest that agents with low levels of conformity (i.e., high self-confidence) have high impacts on group consensus (i.e., enhanced opinion leadership), and Anufriev et al.^27^ show that agents with lower sensitivity to disagreement with others exert more influence on group consensuses. Similarly, Zafeiris and Vicsek^40^ demonstrate that collective decisions are optimal when the pliancy and competence of group members are negatively correlated, implying that the stubbornness of incompetent members contributes to further deviation. The stubbornness of agents in those studies functions in a similar way to the centralities of the agents in the sense that stubborn agents exert disproportionate influence on group consensuses, increasing the deviation. Stubborn agents can be viewed as those having no incoming edges at all (i.e., not being influenced by others) or much fewer incoming edges than outgoing edges (i.e., influencing others more than they are influenced by others) in communication networks, thus exhibiting higher centralities and exerting greater influence on group consensuses than others. To examine the isolated impacts of naïve learning in comparison to those of selective exposure, we exclude confounding factors that could undermine the quality of collective decisions. Specifically, we assume that edges from information sources to recipients are formed *uniformly* at random as in the Erdős-Rényi random graphs^41^ and that all agents are *equally* responsive to the discrepancies with their information sources^42^ (i.e., the absence of both “excessively” influential and “stubborn” agents). Under this assumption, naïve learning should result in a group consensus that is as accurate as the collective decision made by a simple aggregation of opinions.

We then incorporate *selective exposure* into our model as an alternative means for agents to minimize the discrepancies between their own opinions and those of their information sources. In previous literature, “selective exposure” refers to the tendency of people to seek out information that supports their existing views and opinions while avoiding information that challenges their own perspectives^43^. This tendency is commonly observed and has been studied in a wide range of domains, including communication^44^, information processing^45^, attitude and belief formation^46^, as well as decision making^47^. Further, selective exposure has been viewed as a major driving force in the formation and evolution of social networks^48–51^. In the context of collective opinion dynamics, some previous studies formulate selective exposure by assigning differential weights to edges in proportion to the opinion similarity between agents (e.g., interaction frequency^52^, relational intensity^53^, allocated attention^54^, and incentives for homophilic edges^19^), while assuming the connectedness of networks to be exogenously determined and constant over time. However, when the network of agents remains strongly connected, the effects of selective exposure on opinion dynamics are highly limited. Although disagreement among agents is observed temporarily and the convergence of opinions is significantly delayed, groups *always* reach consensus regardless of the level of selective exposure^52,53,55^. In our model, in contrast, connections between agents are assumed to be binary rather than weighted and can be either formed or removed by agents themselves, allowing for endogenous changes in network structure. Specifically, agents can reconstruct their information pools by *removing* the connections to the information sources whose opinions differ most from their own and *adding* new connections to others whose opinions are more congruent with their own than the removed ones. By doing so, agents can reduce the discrepancies with their information sources without adjusting their own opinions.

When endogenous changes in network structure are permitted, an immediate consequence of selective exposure is the fragmentation of a group into small and exclusive subgroups of like-minded agents^54,56,57^, or *echo chambers*, each of which converges to its own unique consensus^27,53^. Whereas previous studies focus on the conditions for fragmentation^57^, the stability of the resulting fragmented structures^19^, the impacts of fragmented structures on the speed of convergence^53^, and the opinion diversity within groups^53,58^, there has been limited research that systematically investigates the effects of a fragmented structure on the quality of collective decisions. Instead, many studies view fragmented or polarized structures as inherently harmful and so as something to be “overcome” through various intervention measures^19,56,57^. Many political researchers, in particular, perceive selective exposure as the primary culprit of political polarization, social distrust and conflict, and intergroup hostility^59–62^. Furthermore, they argue that it reduces the chance for individuals to learn from diverse views, resulting in biased and poor collective decisions^63,64^. From this perspective, political researchers have suggested “cross-cutting exposure” (i.e., the exposure to and learning from diverse views) as a potential solution to these problems^61,65,66^.

Contrary to this near-unanimous belief prevailing in political research and other areas, fragmented structures—more generally *modular* structures—in which individuals are tightly connected to others with similar traits and form cohesive local clusters, are known to be beneficial for maintaining diversity and enhancing groups’ ability to survive and adapt in hostile and uncertain environments^7,58,67–72^. Along these lines, Kao and Couzin^39^ investigate the effects of modular structures on collective decisions, although neither opinion updates nor endogenous changes in network structure are taken into account. The authors stress that the marginal improvement of collective accuracy diminishes, because the amount of information available within a group becomes saturated as its size increases. Therefore, the group size required to achieve a certain level of collective accuracy (i.e., “effective group size”) is typically smaller than the actual group size. Especially when individual opinions are correlated with one another, a modular structure enables a large group to behave as effectively as smaller groups, while retaining other benefits of its large group size. In addition, minority opinions, which might otherwise be overlooked when opinions are simply aggregated, can be better represented within a modular structure, thereby upholding opinion diversity at the global level. Pescetelli et al.^73^ provide empirical evidence directly supporting this view, showing that modular groups composed of small independent subgroups consistently outperform non-modular groups in real-world forecast problems. Along with this line, Mann and Woolley-Meza^7^ underscore the importance of modular structures in retaining intellectual diversity and fostering the generation of new knowledge within academic communities. This can be achieved by maintaining a basic level of isolation and independence between subfields and thereby preventing the dominance of specific models and methods. Adopting Kao and Couzin’s^39^ concept of “effective group size,” we assess the quality of collective decisions achieved under different dynamics against the collective accuracy that a group of the *same size* would have achieved in the absence of social influence among group members.

We perform a series of numerical experiments focusing on the comparative statics of different levels of propensities for selective exposure with respect to group structure, opinion diversity, and the qualities of individual and collective decisions. The numerical experiments yield qualitatively consistent results across all the different settings examined. First, selective exposure fosters exclusive subgroups of like-minded individuals, leading to the emergence of modular and homophilic structures, confirming the findings of previous studies. Also, under strong propensities of selective exposure, opinion diversity *within* subgroups is completely eradicated as each subgroup converges to a local consensus that differs from those of other subgroups, as shown in previous studies. However, opinion diversity at the *global level* is perfectly preserved, suggesting that the diversity-reducing effects of social influence are effectively confined within each subgroup, with no further spread beyond it. Most importantly, the preserved opinion diversity, in turn, maintains the quality of collective decisions at a level as high as that achieved by a simple aggregation of initial opinions. In contrast, naïve learning allows a group to stay connected and thereby reach a consensus that turns out to be more accurate than initial individual opinions, as predicted by previous studies. However, when the effects of group size on the marginal improvement in collective accuracy are taken into consideration, the group consensus is significantly less accurate than both collective decisions made by a simple aggregation and those made under strong propensities for selective exposure across all the simulation settings. This is because naïve learning eradicates opinion diversity at the global level at a faster rate than that of the improvement of individual opinions. That is, the improvement of individual opinions through naïve learning comes at the expense of opinion diversity and collective accuracy, illustrating the trade-off between individual and collective performances.

A unique contribution of the current study is that it examines the causal path from *selective exposure* to the *quality of collective decisions* through the changes in *group structure* and *opinion diversity* in comparison to an alternative mechanism, naïve learning. The causal path is identified based upon a framework that integrates previously disparate concepts and findings, although none of them were completely unprecedented in previous literature. Despite the simplicity of our models, numerical experiments successfully replicate previous findings, indicating that the results we present do not contradict the findings of previous studies. Instead, the current results complement previous studies, providing a new insight into the understanding of the role that selective exposure plays in collective decision-making, which has been often perceived as a frustrating obstacle to achieving collective intelligence. The implication of the current results is straightforward: A fragmented group structure resulting from selective exposure prevents *both* information and influence from flowing across subgroups, while naïve learning allows *both* to flow. This is because communication channels between agents cannot separate information from the influence it carries. In that sense, the current findings simply suggest that blocking both information and influence (i.e., preserving diversity at the expense of additional information) is more beneficial for collective decisions than allowing both to flow (i.e., gaining additional information at the cost of opinion diversity), reconfirming the crucial role of opinion diversity in collective decision-making.

The rest of this paper is organized as follows. The next section introduces the agent-based models examined in this study, with detailed descriptions of simulation settings and measures. The results from numerical experiments are then summarized in the subsequent section. The paper concludes with a discussion of the main findings and proposals for future research.

## Methods

### Classical models of collective decision-making

The conditions for achieving collective intelligence have been extensively studied. One of the earliest models is Condorcet’s jury theorem^23^. The theorem considers a group of *n* members tasked with deciding between two alternatives, one of which is objectively better than the other but unknown to the members. Each member is assumed to choose the better decision with a probability of $p>.5$, which is at least slightly better than a random guess. Further, all decisions are assumed to be made independently and then aggregated into the group’s decision by the majority rule. Under these assumptions, the probability of a group arriving at the correct decision ${P}_{G}$ is always greater than individual competence $p$. Moreover, ${P}_{G}$ increases monotonically and approaches 1 in the limit as group size increases.

Galton’s field experiment^24^ provides similar results. In his experiment, participants were asked to guess the weight of a live ox, and the median estimate of valid guesses (i.e., “valid” in the sense of being uninfluenced by others’ guesses) was accurate within a 1% margin of the ox’s true weight. Later, he found the mean estimate even more accurate^25^. Based on these observations, he argued that a simple aggregation of independent judgments can produce a sufficiently accurate collective outcome because the errors in individual judgments tend to cancel each other out when aggregated. Also, it can be intuitively conceived that the errors in collective outcomes decrease as group size increases, as indicated by the central limit theorem.

Even though Condorcet and Galton consider different types of opinions (i.e., binary or continuous opinions) and different methods for aggregating individual decisions (i.e., the majority rule or the median and mean aggregation), they both reach the same conclusion: Groups outperform individuals in decision-making, and the quality of collective decisions monotonically increases as a function of group size (see Section S1 in *Supplementary Materials* for more details). However, both share the unrealistic assumption that individuals in groups make their decisions *independently* without influencing or being influenced by others.

To formulate a model of opinion dynamics under social influence, DeGroot’s model^26^ assumes that individuals update their opinions by repeatedly adopting the average of others’ opinions, which is formulated as a Markov process as follows. Individual opinions $\mathbf{x}\left(t\right)$ at a time point $t\in \left\{1, 2, 3, \dots \right\}$ are determined as the weighted means of others’ opinions at $t-1$:

$$\mathbf{x}\left(t\right)=\mathbf{W}\mathbf{x}\left(t-1\right)\text{ or }\mathbf{x}\left(t\right)={\mathbf{W}}^{t}\mathbf{x}\left(0\right),\text{ where }\mathbf{W}=\left[{w}_{ij}\right],$$

where ${w}_{ij}$ denotes the relative influence of *j*’s opinion on the update of *i*’s opinion (i.e., ${0\le w}_{ij}\le 1$), and **W** is a row-stochastic transition matrix (i.e., ${\sum }_{j}{w}_{ij}=1$ for all $i$) that can be interpreted as the adjacency matrix of the network that describes the patterns of social influence among individuals in a group. Under these dynamics, individual opinions $\mathbf{x}\left(t\right)$ converge to a steady-state distribution ${\mathbf{x}}^{\boldsymbol{*}}$ in the limit:

$${\mathbf{x}}^{\boldsymbol{*}}=\underset{t\to \infty }{{\text{lim}}}\mathbf{x}\left(t\right)=\underset{t\to \infty }{{\text{lim}}}{\mathbf{W}}^{t}\mathbf{x}\left(0\right)={\mathbf{v}}_{1}\mathbf{x}\left(0\right),$$

where ${\mathbf{v}}_{1}$ is the eigenvector corresponding to the leading eigenvalue of **W**.

The theorem identifies the two conditions under which a group reaches a consensus (i.e., all the entries of ${\mathbf{x}}^{\boldsymbol{*}}$ become equal). First, the Markov process is capable of transitioning from any state to any other state with a non-zero probability, having no isolated subset of states (i.e., irreducibility). Second, the process does not exhibit a regular, repeating pattern in its transition (i.e., aperiodicity). Put simply, the group *always* reaches a consensus, so far as the influence network remains strongly connected^33^. Further, the entries of ${\mathbf{v}}_{1}$ are equivalent to the eigenvector centralities of corresponding agents in the network^32^. Thus, the group consensus ${\mathbf{v}}_{1}\mathbf{x}\left(0\right)$ is not necessarily the “middlemost” opinion that evenly reflects the diverse opinions initially held by individual members, and thus the errors in initial opinions cannot be effectively canceled out when aggregated. Instead, the group consensus is determined—or at least, highly influenced—by a few individuals who occupy the most central positions in the group, which necessarily results in a deviation from the collective decision formed by a simple aggregation and the true state of nature.

However, the introduction of selective exposure into opinion dynamics will prevent a group from reaching a global consensus. This is because selective exposure results in the fragmentation of the group into smaller subgroups (i.e., strongly connected components). In that case, the conditions for reaching a *global* consensus (i.e., irreducibility and aperiodicity) will no longer be met. Yet, each subgroup independently reaches a distinct *local* consensus. In this way, opinion diversity can be preserved at the global level.

### Agent-based models

We extend DeGroot’s model by allowing endogenous changes in networks through selective exposure and then examine its impacts on group structure, opinion diversity, and the quality of collective decisions in comparison to naïve learning. To this end, we consider four agent-based models, including *Condorcet* and *Galton Models*, which simulate the settings depicted in Condorcet’s theorem and Galton’s experiment, respectively, and *Bimodal* and *Exponential Models*, which simulate initially polarized and skewed opinion distributions, respectively. The aspects shared by all these models are described first and followed by the details specific to each model, including the distributions of initial opinions, the rules used to aggregate individual decisions into collective decisions, and the expected qualities of the collective decisions formed in the absence of social interactions, which are to be compared against the simulation results from each model.

#### Common settings

All four models consider a group of *n* agents. Each agent starts with its own opinion ${x}_{i}$. The initial opinion ${x}_{i}$ is an *i*.*i*.*d*. random variable that follows a probability distribution specified in each model. This ensures that every agent begins with an independent opinion, and so the group starts with sufficient opinion diversity. Also, each agent is initially connected to *k* other agents that serve as its information sources. The connections between agents are directed and not necessarily reciprocal. The number of connections $k$ is constant and finite, following the assumption that individuals have a limited capacity of interactions^18,19,52,54^. The initial connections between agents are formed uniformly at random and independent of their initial opinions, ensuring that agents are exposed to diverse opinions at the beginning. The communication network of agents *G* is a random directed graph with a fixed in-degree*,* and its adjacency matrix ${A}_{ij}$ represents the directions of information flow from agents *i* to *j*. However, out-degrees follow a binomial distribution as in the Erdős–Rényi random graph model^41^. The set of agent *i*’s information sources, or its “information pool,” is denoted by $G\left(i\right)=\left\{j \right| {a}_{ij}=1\}$.

In each round of simulations, agents attempt to minimize the discrepancies between their own opinions and those of their information sources in a random order, following the models used in previous studies^30,42^. *Discrepancy* is measured as the sum of differences between an agent’s opinion and those of its information sources ${\sum }_{j\in G\left(i\right)}\left|{x}_{i}-{x}_{j}\right|$ (i.e., the sum of absolute deviations). We consider two ways of reducing discrepancy. The first is *naïve learning*, by which an agent simply adopts the opinion ${x}_{i}^{\prime}$ such that it minimizes the discrepancies with its information sources:

$${x}_{i}^{\prime}={\text{argmin}}\sum_{j\in G\left(i\right)}\left|{x}_{i}-{x}_{j}\right|.$$

whereas previous studies assume agents to adopt the “mean” opinion of others, we assume agents to adopt the “median” opinion in their information pool for several reasons. First, when the amount of discrepancy is measured as the sum of “absolute” deviations rather than “squared” deviations, the median minimizes discrepancy^74^. Second, the statistical behavior of sample median is asymptotically equivalent to that of sample mean, regardless of the original distribution from which a sample is drawn^75^. Third, when the distribution of initial opinions is skewed, the median is robust against outliers^76,77^, producing more stable results. Fourth, the “mean” opinion is obtained by the linear combination of others’ opinions, which makes mathematical analysis convenient and allows for straightforward calculations and interpretation. However, using the “mean” opinion drastically increases the computational time for a simulation to reach its steady state. In theory, no simulation of DeGroot’s model can reach its steady state within a finite amount of time. Even when an arbitrary stopping rule is applied, a simulation can approach “closely enough” to its steady state at best. On the other hand, when the “median” opinion is used, simulations can be completed within a reasonable amount of time, while generating practically identical and stable results.

Alternatively, agents can reduce discrepancy via *selective exposure*, by which an agent removes its connection to the information source whose opinion differs most from its own and adds a new connection to a randomly selected agent whose opinion is more congruent with its own than the removed one. In each round, each agent chooses either selective exposure with a probability of $\beta$ or naïve learning with its complement of $1-\beta$. A set of 51 evenly spaced values between 0 and 1 is used to quantify the parameter describing agents’ propensity for selective exposure: $\beta \in \{.00, .02, .04., \dots , 1.00\}$. For each value of $\beta$, a total of 30,000 simulations are performed.

#### Model specific settings

**Figure 1:**
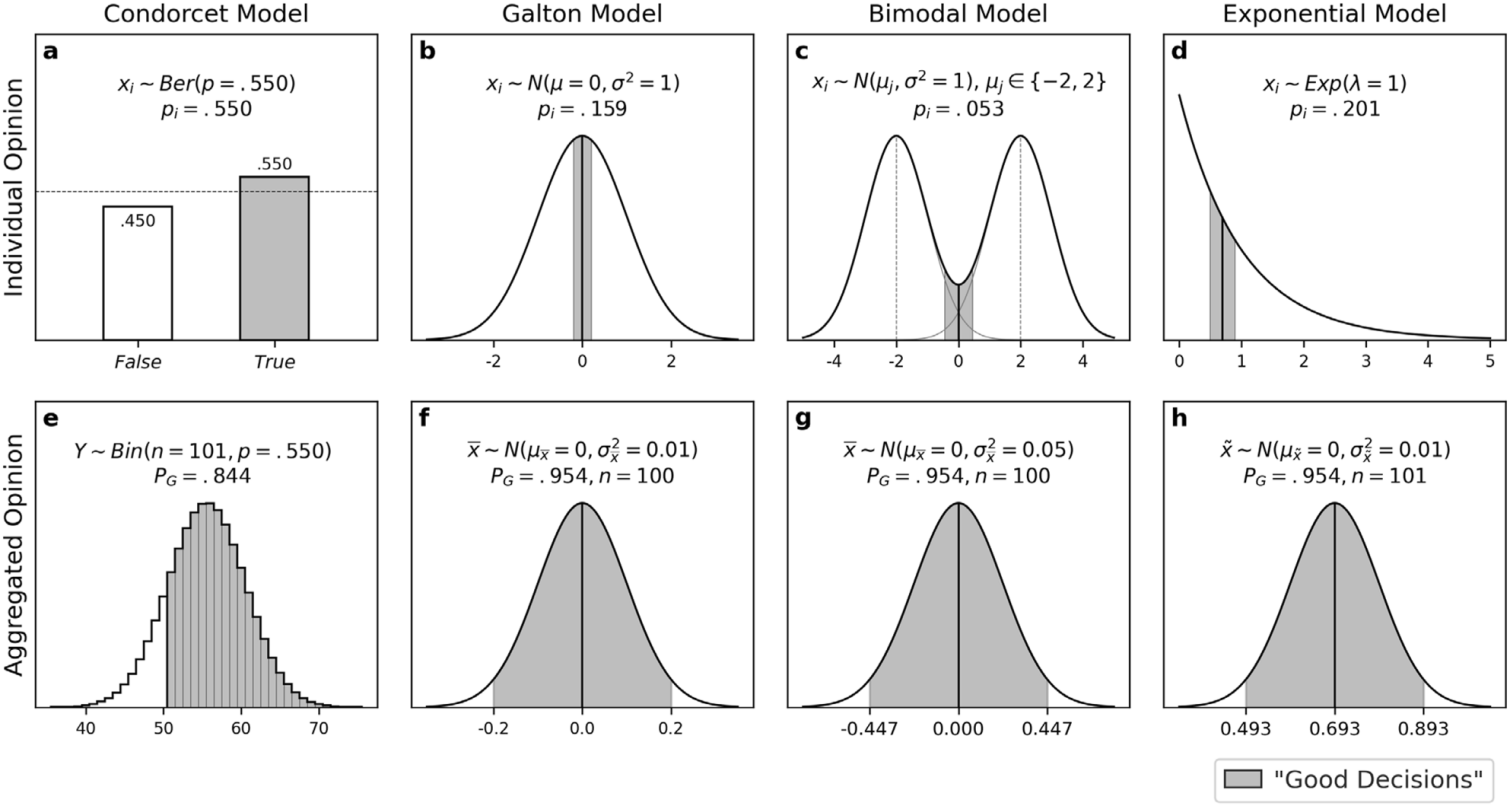
The distributions of initial individual opinions (**a** through **d**) and aggregated initial opinions (**e** through **h**). The shades indicate “Good Decisions,” operationally defined as *True* decisions for the Condorcet model and as those that fall within a range of two standard errors from the true state of nature in the other models.

First, group sizes in the *Condorcet Model* are set as an odd number, $n=101$, to avoid the need for tie-breaking rules. Adopting the assumption of Condorcet’s theorem, the initial individual opinion ${x}_{i}\in \left\{True, False\right\}$ for $i=1, 2, 3, \dots , n$ is an *i*.*i*.*d* Bernoulli random variable, and individual competence is set as $p=.55$ (Fig. 1a). When initial opinions are aggregated, the number of correct decisions ${\sum }_{i}^{n}{x}_{i}$ in a group follows a binomial distribution $y={\sum }_{i}^{n}{x}_{i} \sim Bin\left(n=101,p=.55\right)$. In that case, the probability of the group reaching the correct decision by the majority rule is defined as the sum of probability mass $P\left(Y>n/2\right)=.844$ (Fig. 1e).

Second, group sizes in the *Galton Model* are set as $n=100$ and the initial individual opinions as an *i*.*i*.*d* standard normal random variable ${x}_{i} \sim N(\mu =0, {\sigma }^{2}=1)$, where the true state is $\mu =0$ (Fig. 1b). The collective decision $\overline{x }$ is determined as the mean of individual opinions. Thus, collective decisions follow a normal distribution with mean $\mu =0$ and variance ${\sigma }_{\overline{x} }^{2}=0.01$ by the central limit theorem (Fig. 1f): $\overline{x } \sim N(\mu =0, {\sigma }_{\overline{x} }^{2}=0.01)$.

As an extension of the Galton Model, we consider the *Bimodal Model* in which initial individual opinions follow a mixture of two normal distributions, both sharing a common variance but having different means, such that the overall distribution has two peaks (Fig. 1c). This model is intended to reflect the polarized opinion distributions commonly observed in political contexts^78,79^. To generate the initial opinion ${x}_{i}$, a local mean ${\mu }_{j}$ is chosen between $-2$ and $2$ with an equal probability, and then, a normal random variable with local mean ${\mu }_{j}$ and common variance ${\sigma }_{j}^{2}=1$ is generated:

$${\mu }_{j} \sim Ber\left(q\right)\text{ and }{x}_{i} \sim N({\mu }_{j}, {\sigma }_{j}^{2}),\text{ where }q=.5, {\mu }_{j}\in \left\{-2, 2\right\},\text{ and }{\sigma }_{j}^{2}=1.$$

The distribution of initial individual opinions is symmetric around the global mean $\mu =0$ ($=q{\sum }_{j=1}^{2}{\mu }_{j}$) and has two peaks at the local means. By the law of total variance, the variance of the distribution is ${\sigma }^{2}=5$:

$${\sigma }^{2}=\sum_{j=1}^{2}q{\left({\mu }_{j}-\mu \right)}^{2}+\sum_{j=1}^{2}q{\sigma }_{j}^{2}.$$

Group size is set as $n=100$, and collective decisions are represented by the mean of individual opinions as in the Galton model. Under the condition of independence, collective decisions are normally distributed with mean $\mu =0$ and variance ${\sigma }^{2}=0.05$ by the central limit theorem (Fig. 1g): $\overline{x } \sim N(\mu =0, {\sigma }_{\overline{x} }^{2}=0.05)$.

Lastly, the *Exponential Model* assumes that initial individual opinions are highly skewed, following an exponential distribution with rate parameter $\lambda =1$ (Fig. 1d). Unlike the previous models, collective decisions are represented by the *median* of individual opinions (i.e., median aggregation). Because the median is robust against outliers, it is the preferred measure of central tendency for skewed distributions^76,77^. Accordingly, the true state of nature is set as the median of the distribution, $\widetilde{\mu }={\text{ln}}2/\lambda =0.693$.

The statistical behavior of sample median $\widetilde{x}$ is known as follows^75^: When a sample of *n* random variables $\{{x}_{1}, {x}_{2},\dots , {x}_{n}\}$, where $n=2m+1$ and $m$ is a positive integer, are independently and identically drawn from a probability distribution with a density function $f(x)$, the distribution of sample medians is approximately normal with mean $\widetilde{\mu }$ (i.e., the median of the parent distribution) and variance $\frac{1}{8f{\left(\widetilde{\mu }\right)}^{2}m}$ for large *n*. To utilize this statistical property of sample median, we set group size as $n=101$ (or $m=50$). In that case, collective decisions approximately follow a normal distribution with mean $\widetilde{\mu }={\text{ln}}2=0.6931$ and variance 0.01, as $f\left(\widetilde{\mu }\right)={e}^{-{\text{ln}}2}=1/2$ and $8f{\left(\widetilde{\mu }\right)}^{2}m=8\cdot {\left(1/2\right)}^{2}\cdot 50$ (Fig. 1h).

In addition to the results presented below, we also examined each of the four models under different parametric settings to establish the robustness of our results. These additional settings include different initial network structures (i.e., scale-free and regular networks), numbers of information sources ($k=3$ and $k=9$), and group sizes ($n=400$ for the *Galton* and *Bimodal Models* and $n=401$ for the *Condorcet* and *Exponential Models*). These results are presented in *Supplementary Materials *(Section S2), where they can be seen to reproduce qualitatively similar outcomes.

#### Measures

Each simulation is terminated and considered to have arrived at its steady state when no further change occurs either in connections between agents or in their opinions. Individual opinions at steady states are considered as the final decisions that individual agents eventually arrive at, denoted by ${x}_{i}^{*}$, and then aggregated to form collective decisions, denoted by ${\overline{x} }^{*}$ (or ${\widetilde{x}}^{*}$ for the Exponential Model).

In the *Condorcet Model*, *individual performance* ${p}_{i}$ is measured as the proportion of agents whose final decisions are $True$. Similarly, *collective performance* ${P}_{G}$ is measured by the proportion of groups whose majority decide $True$ at steady states. Given that group size $n=101$, it is expected that ${p}_{i}=.55$ and ${P}_{G}=P\left(\frac{1}{n}{\sum }_{i}^{n}{x}_{i}^{*}>.5\right)=.844$ in the absence of social influence.

To measure individual and collective performances in the other models that assume continuous opinions, we first operationalize “Good Decisions” and then measure individual performance ${p}_{i}$ and collective performance ${P}_{G}$ as the fractions of “good decisions” found at steady states. “Good decisions” are defined as those that fall within a range of two standard errors from the true state of nature, adopting the conventional decision criterion in statistical analysis. That is,

$$Good\, Individual\, Decisions\,\,{H}_{i}=\left\{{x}_{i}^{*} | \left|{x}_{i}^{*}-\mu \right|<2{\sigma }_{\overline{x}}\right\}\,\,and$$

$$Good \,Collective\, Decisions \,\,{H}_{G}=\left\{{\overline{x} }^{*} | \left|{\overline{x} }^{*}-\mu \right|<2{\sigma }_{\overline{x}}\right\},$$

where the standard error is ${\sigma }_{\overline{x} }=\sigma /\sqrt{n}$, and $\mu$ is the true state ($\widetilde{\mu }$ for the Exponential Model) specified by a model. Then,

$$Individual \,Performance\,\,{p}_{i}=\frac{\left|{H}_{i}\right|}{n} \,\,and$$

$$Collective \,Performance\,\,{P}_{G}=\frac{\left|{H}_{G}\right|}{N},$$

where *n* is the group size, and *N* is the total number of simulations for each value of $\beta$.

Previous studies measure collective performance, as alluded to in references such as “wise society”^33^, “wise crowd”^30^ and “(mis)information”^34^, by the (squared) deviation of a collective decision from the true state of nature and show that the deviation approaches zero (i.e., achieving collective intelligence) in the absence of “excessively” influential agents as group size approaches infinity. However, it should be noted that the deviation is inversely proportional to the square root of group size $\sqrt{n}$, and therefore the infinitely large size of groups is sufficient for the deviation to converge to zero, as indicated by the central limit theorem. Also, the sublinear relationship between the deviation and group size demonstrates the diminishing marginal improvement of collective performance, as pointed out by Kao and Couzin^39^. Therefore, when the effects of group size are not properly controlled for, the deviation alone tends to overestimate collective performance and thereby underestimate the negative effects of influential agents. For this reason, we include standard error ${\sigma }_{\overline{x} }$ to define the range of “good decisions,” which allows for direct and more precise comparison between collective performances under different settings across models. This is because the ranges of “good decisions” are all standardized. In the absence of social influence (i.e., a simple aggregation of initial opinions), a group is expected to make a “good” collective decision with a probability of 0.954 $\left[=P\left(\left|{x}_{i}^{*}-\mu \right|<2{\sigma }_{\overline{x} }\right)\right]$, since $({\overline{x} }^{*}-\mu )/{\sigma }_{\overline{x} }$ follows the standard normal distribution $N(0, 1)$, regardless of the distributions of initial opinions. Also, in the case of an infinitely large group, our definition becomes equivalent to those in previous studies $\left\{{\overline{x} }^{*} | \left|{\overline{x} }^{*}-\mu \right|=0\right\}$, since ${\sigma }_{\overline{x} }$ converges to zero.

In addition, we measure the *opinion diversity* in groups ${\sigma }_{x}$, the *size of the giant components* $\left|{C}_{1}\right|$, and the *modularity* $Q$ of networks at steady states to examine the impacts of local dynamics on the global structure. Opinion diversity ${\sigma }_{x}$ is measured by the standard deviation of individual opinions at steady states. The size of the giant component $\left|{C}_{1}\right|$ is measured by the proportion of nodes that belong to the largest strongly connected component of a communication network $G$, within which every node is reachable from every other node. Modularity $Q$ is measured as

$$Q=\sum_{d}\left[\frac{{L}_{d}}{m}-{\left(\frac{{k}_{d}^{{\text{in}}}{k}_{d}^{{\text{out}}}}{2m}\right)}^{2}\right],$$

where $d$ represents the dichotomized categories of individual opinions (i.e., *True* or *False*; *above* or *below* the true state), $m$ is the total number of edges, ${L}_{d}$ is the total number of edges within category $d$, and ${k}_{d}^{{\text{in}}}$ and ${k}_{d}^{{\text{out}}}$ are the sums of in-degrees and out-degrees of the nodes in category $d$, respectively^80^. Both the size of the giant components $\left|{C}_{1}\right|$ and modularity $Q$ are used to measure the degree to which the communication network is fragmented. Low values of $\left|{C}_{1}\right|$ and high values of $Q$ indicate the presence of echo chambers within groups.

## Results

### Sampled results: replication of previous findings

**Figure 2:**
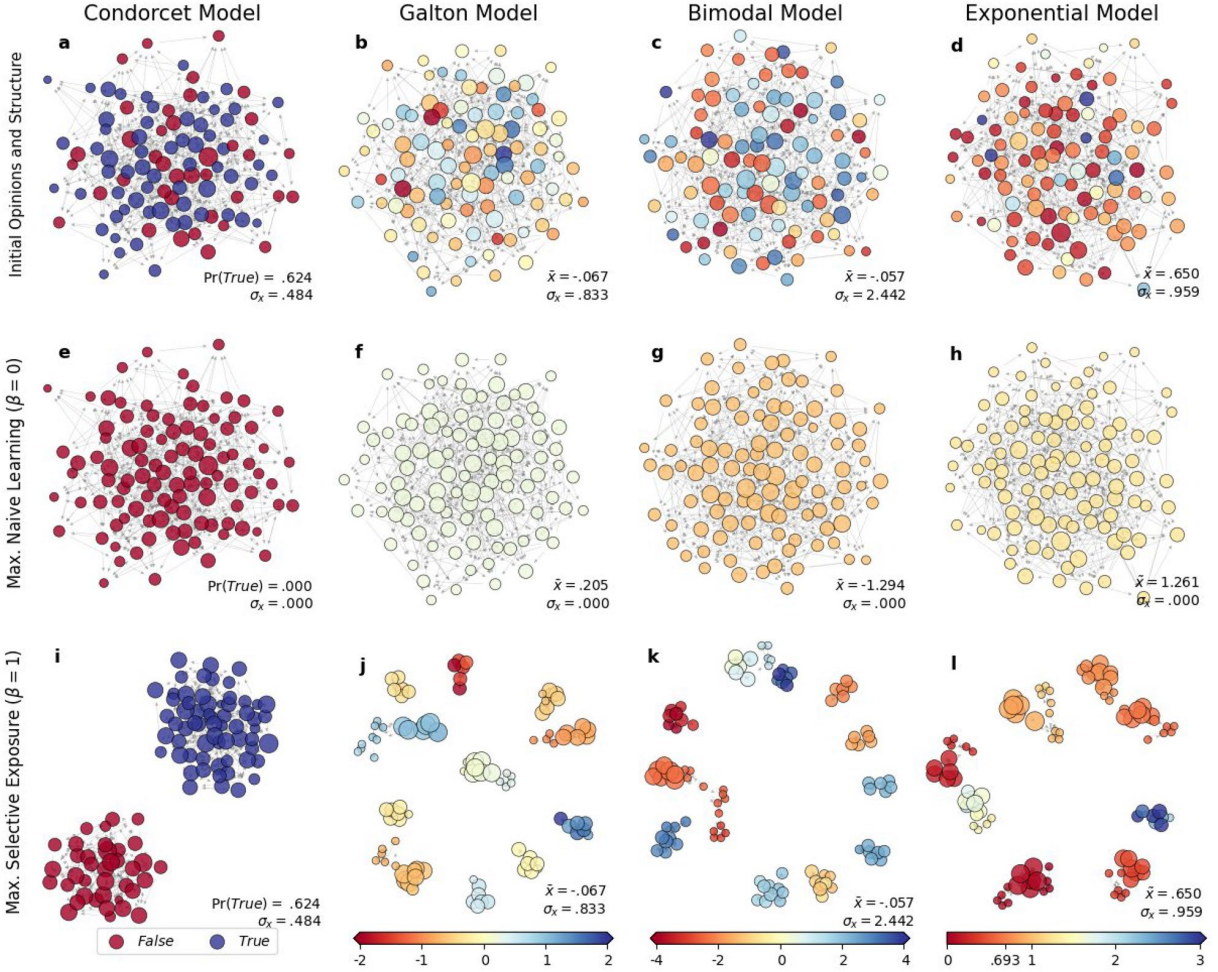
Sampled results from the simulations of the four models: Initial opinion distributions and network structure of groups (**a** through **d**) and those at steady states under the maximal propensities for *naïve learning* ($\beta =0$, **e** through **h**) and *selective exposure* ($\beta =1$, **i** through **l**). Node size and color indicate the eigenvector centralities and the opinions of corresponding agents, respectively.

Figure 2 visualizes the sampled results from numerical experiments and compares opinion distributions and group structures at the initial states with those at steady states under the maximal propensities for naïve learning ($\beta =0$) and selective exposure ($\beta =1$). In each plot, nodes represent individual agents, and their colors and sizes indicate the opinions and eigenvector centralities of the corresponding agents, respectively. Nodes are placed closely to one another if they are connected.

Across all four models, groups initially contain diverse opinions, and agents with different opinions are connected to one another (Figs. 2a–d). However, the two different dynamics—*naïve learning* and *selective exposure*—yield drastically different outcomes. Under the maximal propensity for naïve learning ($\beta =0$, Figs. 2e–h), all agents end up adopting the same opinion, and thus, groups reach a consensus, as DeGroot’s model predicts. As a result, the opinion diversity at the global level vanishes completely (${\sigma }_{x}=0$), demonstrating the diversity-reducing effects of social influence. Under the maximal propensity for selective exposure ($\beta =1$, Figs. 2i–l), agents alter their local networks without adjusting their opinions at all. As a result, agents with similar opinions end up being densely clustered and form exclusive subgroups between which no edges are found. Opinions within each subgroup are nearly identical, indicating the loss of opinion diversity within subgroups. However, each subgroup reaches its own unique consensus distinguished from those reached by others, and therefore, the global-level opinion diversity ${\sigma }_{x}$ is perfectly preserved. These results confirm the findings of previous studies, validating our approaches.

### The effects of selective exposure

**Figure 3:**
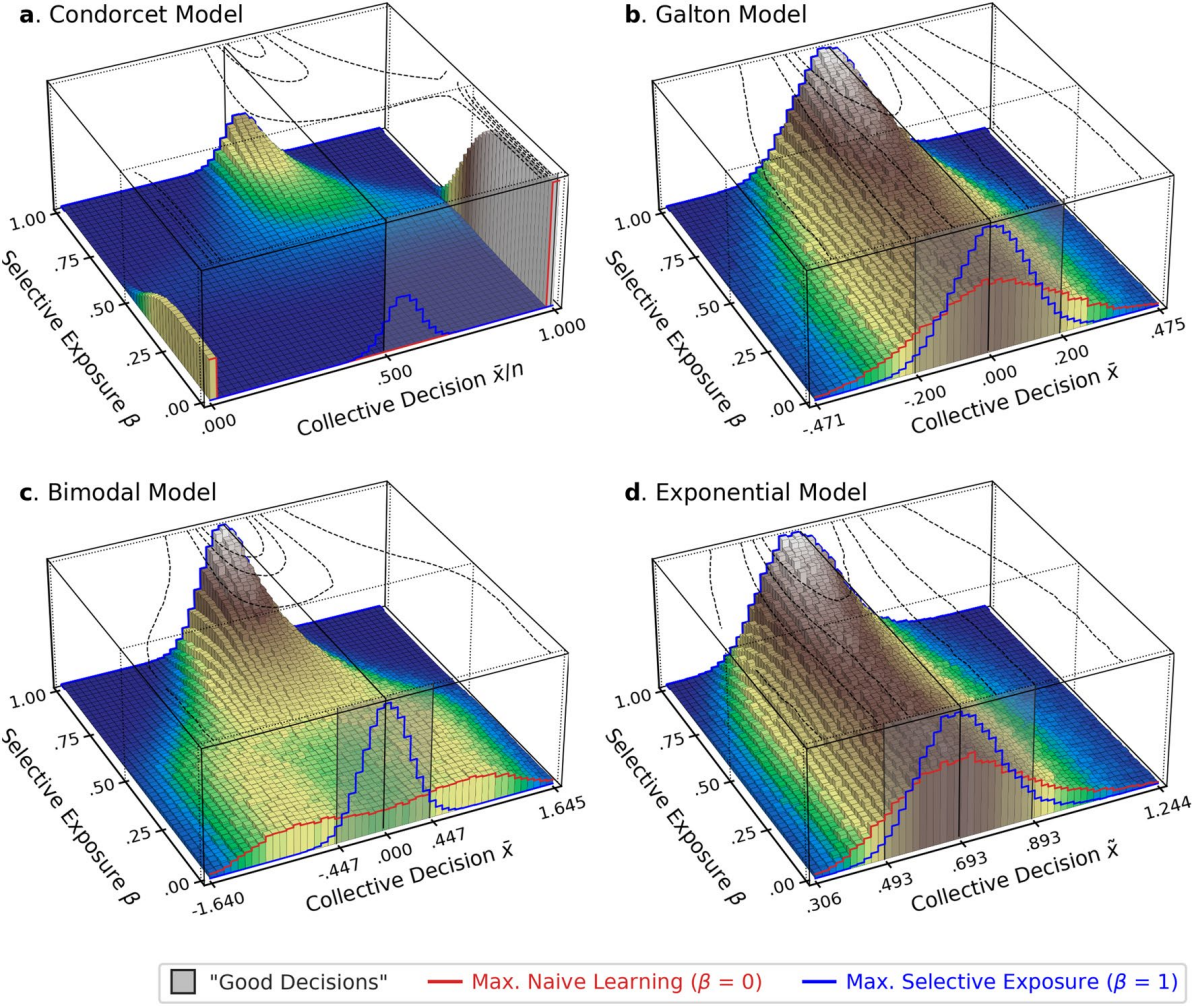
3D histograms of the collective decisions formed at different levels of selective exposure across the four models. The red and blue histograms projected on the front represent the distributions of collective decisions reached under the maximal propensities for *naïve learning* ($\beta =0$) and *selective exposure* ($\beta =1$), respectively.

Figure 3 presents 3D histograms in which the heights of bars represent the relative frequencies of collective decisions obtained from 30,000 simulations for each of the intermediate levels of propensity for selective exposure $\beta \in \left\{.00, .02, .04., \dots , 1.00\right\}$ across the four models.

Across all the models, the distributions of collective decisions formed under the maximal propensity for *selective exposure* ($\beta =1$; the blue histograms projected on the front in Fig. 3) are nearly identical to those made by a simple aggregation of initial opinions (Figs. 1e–h). In terms of collective performance, 84.4% of groups arrive at the correct decision in the Condorcet models, and 95.4% of groups make “good” decisions in the other models, suggesting that collective performance is maintained at a level as high as that achieved by a simple aggregation of initial opinions. On the other hand, as *naïve learning* becomes more dominant, the distributions become more dispersed with longer and thicker tails, indicating that collective decisions are more likely to deviate from the true state. These deviations are most salient in the *Condorcet model* due to the binary nature of the opinions it models (i.e., *True* or *False*). When *naïve learning* is dominant over *selective exposure* ($\beta <.50$), groups produce only two collective outcomes: Either all agents make the correct decision, or all make the wrong decision. Effectively, each group is constrained to behave like a single individual.

Although their results are not as extreme as those observed in the Condorcet Model, similar patterns are observed in the other models that assume continuous opinions. In the *Bimodal Model*, when *naïve learning* is dominant, the distribution of collective decisions resembles that of initial individual opinions characterized by the two peaks, implying that collective decisions are effectively determined by a few agents who happen to occupy central positions in the communication network and so exert more influence on the collective decisions. In the *Exponential Model*, collective decisions reached under the maximal propensity for *naïve learning* are skewed to the right, reflecting the initial distribution of individual opinions, even though the method of median aggregation is robust against outliers. This also illustrates that collective decisions tend to be determined by a few central agents when *naïve learning* is dominant.

**Figure 4:**
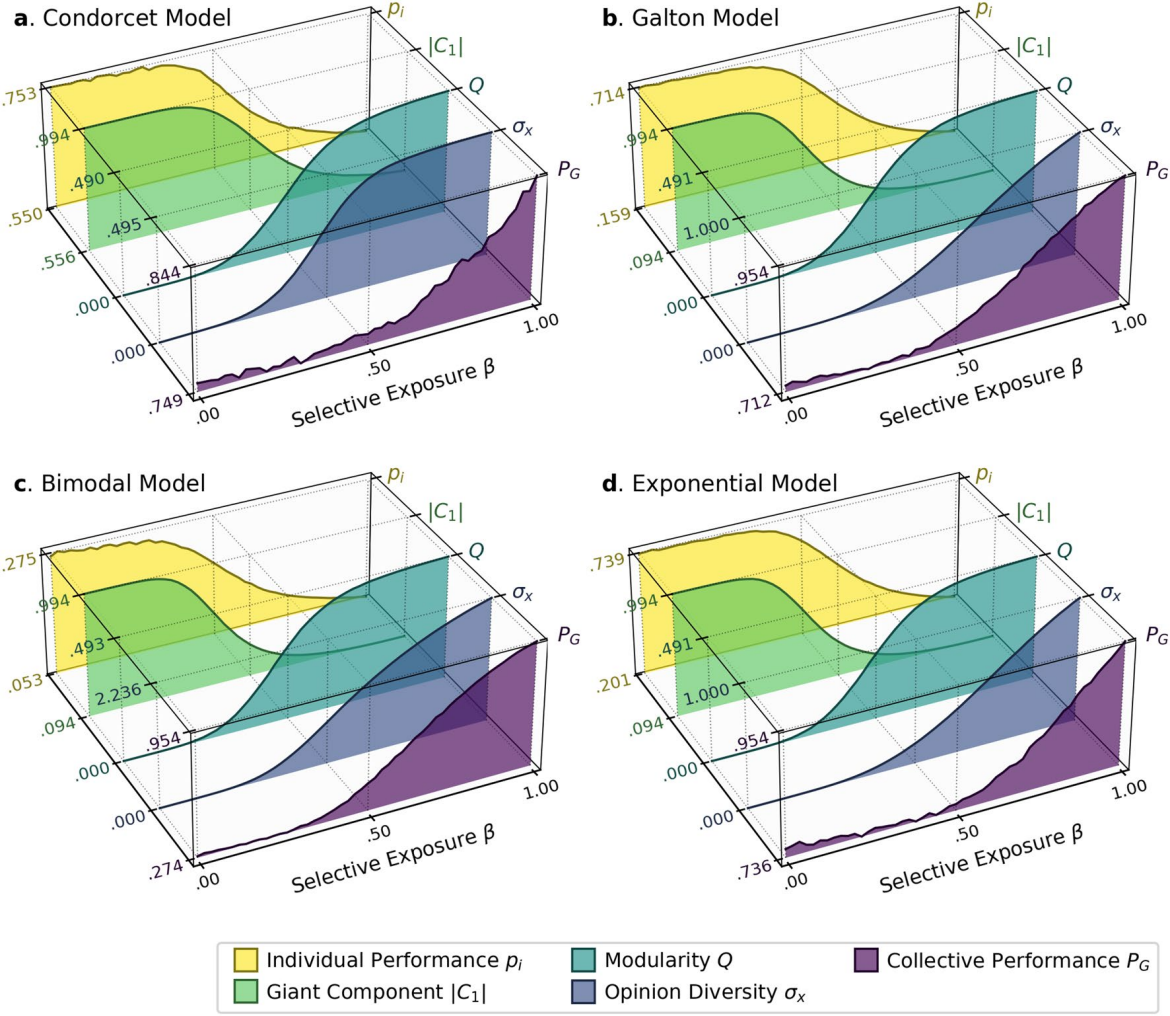
The effects of selective exposure on individual performance ${p}_{i}$, giant component size $\left|{C}_{1}\right|$, modularity $Q$, opinion diversity ${\sigma }_{x}$, and collective performance ${P}_{G}$.

Figure 4 summarizes individual performance ${p}_{i}$, giant component size $\left|{C}_{1}\right|$, modularity $Q$, opinion diversity ${\sigma }_{x}$, and collective performance ${P}_{G}$ at different levels of *selective exposure*, each of which displays nearly identical patterns across all the four models. First, selective exposure sharply decreases the size of the giant component $\left|{C}_{1}\right|$ and increases modularity $Q$, which together suggest that selective exposure leads to the emergence of exclusive and disconnected subgroups of like-minded individuals. Second, selective exposure preserves opinion diversity ${\sigma }_{x}$ at the global level, suggesting that the diversity-reducing effect of social influence has been effectively confined within subgroups. Most importantly, selective exposure allows groups to maintain their collective performance at a level as high as that achieved by a simple aggregation of initial opinions, as already shown in Fig. 3.

One important observation is that whereas individual performance ${p}_{i}$ remains unchanged under strong propensities for *selective exposure*, it is substantially improved under strong propensities for *naïve learning* across all the models (the yellow layers in Fig. 4), which is consistent with previous findings^33,34^. For instance, when agents naïvely adopt the majority opinion in their information pools, 75.3% of agents end up making the correct decision at the steady states in the Condorcet Model (${p}_{i}=.753$ when $\beta =0$). This proportion is substantially higher than the initial individual competence $p=.550$, which can be explained as follows. A naïve learner adopts the “majority” or “median” opinion of its information pools. Therefore, the updated opinion is effectively a “collective” decision made by simply aggregating the opinions of its information sources, as in Condorcet’s theorem and Galton’s experiment. Even though the size of this “group” is small ($k=5$ in our simulation settings), the updated opinion should be statistically more accurate than any of the individual opinions held by one’s information sources. In that sense, individual opinions are expected to progressively improve through each round of naïve learning.

Contrary to the findings of previous studies^33,34^, however, collective decisions formed through naïve learning significantly deviate from the true state *despite* the improvement in individual decisions (the purple layers in Fig. 4). This cannot be attributed to the presence of “excessively” influential agents, which is unlikely in our models because all the connections between agents are formed *uniformly* at random. Instead, the current results suggest that the negative effects of (even “moderately”) influential agents are much greater than predicted by previous studies (see also Section S2.3 in *Supplementary Materials*). Further, the lower collective accuracy, despite improved individual accuracy, suggests the importance of opinion diversity for collective decision-making relative to individual competence. Naïve learning, operating as collective decision-making at a small and local scale, progressively improves individual opinions, but the rate of improvement drastically decreases because the opinions within information pools become homogenous over time at a faster rate than that of improvement. At steady states, the aggregation of opinions in one’s information pool cannot yield any improvements in collective accuracy, simply because not only the information sources but also all other agents adopt a common opinion. That is, the whole group effectively behaves as if it *were* a single “moderately competent” individual. According to our simulation results, for example, individual agents make the correct decisions with a probability of 0.753 under the maximal propensity for naïve learning in the Condorcet Model (${p}_{i}=.753$), while collectively making the correct decisions with a probability of 0.749 (Fig. 4a). The minor gap between the two probabilities is due to the failure of groups to remain strongly connected and thus to reach consensus [i.e., $P\left(\left|{C}_{1}\right|<1\right)=1-.994$]. Either probability is significantly lower than the probability of a group making the correct decision by a simple aggregation of initial opinions (0.844 in Fig. 1e). Not surprisingly, similar patterns are observed in the other models.

The results presented above are all successfully reproduced in extended numerical experiments performed over a wider range of simulation settings, confirming the robustness of the results above (see *Supplementary Materials* Section S2 for more details). In addition, the results from the extended numerical experiments allow us to identify the conditions under which naïve learning can also function to maintain the quality of collective decisions despite the utter absence of opinion diversity within groups. First, when each agent has access to a *large pool of information*, both individual and collective decisions improve (Section S2.2). Ideally, if every agent can be exposed to and has a chance to learn from everyone else’s opinions (i.e., $k=n-1$; complete networks), collective decisions under naïve learning are expected to be as good as those made under the maximal propensity for selective exposure or in the absence of social influence. Second, the group structure should be perfectly *decentralized* (i.e., regular networks). That is, every group member must exert exactly an equal amount of influence on everyone else’s decision, and there must be neither “leaders” nor “followers” in the group (Section S2.3). Although these conditions can be implemented in our agent-based models, they are hardly feasible or expected to be met in real-world collective decision-making.

### Collective decision-making under time constraints

**Figure 5:**
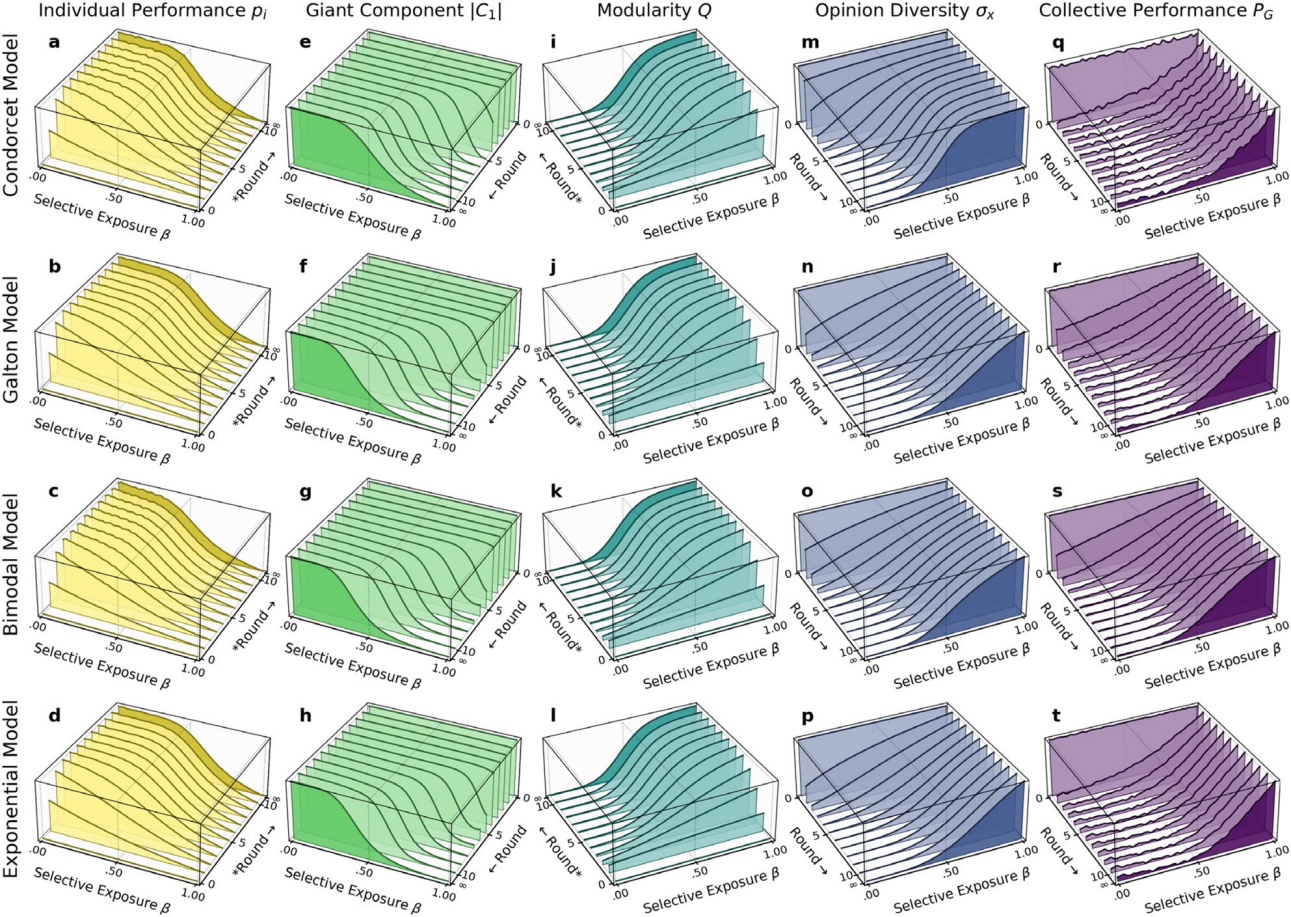
Changes in performance and group structure for the first ten rounds at different levels of propensity of selective exposure. The darkened layer in each plot represents the corresponding measures at steady states. *Note*: “Rounds” for *individual performance* ${p}_{i}$ and *modularity* $Q$ are arranged in reverse order for a clearer visual presentation.

In real-world decision-making, both individual and collective decisions are typically subject to time constraints. Rather than having unlimited time to deliberate, individuals and groups are often forced to make decisions *before* arriving at a steady state. In that sense, transient opinion dynamics may be more relevant. From this perspective, we examine the effects of selective exposure and naïve learning during the early stages of dynamics (Fig. 5).

Figure 5 visualizes the changes in performance and group structure for the first ten rounds, during which most major changes occur. Even after the very first round, selective exposure (as well as naïve learning) already shows non-trivial impacts on both performance and group structure. Subsequently, all the measures remain nearly the same until steady states are reached (the darkened layer in each plot). These results suggest that the effects of selective exposure become observable almost immediately, further reinforcing the robustness of our findings. Even if decisions must be made over truncated time scales, selective exposure still affects both individual and collective performances and group structure in a manner consistent with the steady-state results reported above. It should be noted that these “immediate” effects observed in the early stage of simulations are “relative” to the time scale, and thus they can be seen as an artifact of the parameter settings specified in our numerical experiments.

Furthermore, the changes observed over consecutive rounds help explicate the causal relationships among the local behavior of agents, global structural properties, and individual and collective performances, allowing for a better understanding of the steady-state results reported above. Naïve learning substantially improves individual performance for the first few rounds (the first column in Fig. 5), as agents update their opinions by aggregating diverse opinions in their information pools (i.e., collective decision-making at small and local scales), but the rate of improvement sharply declines. This is because the repetition of naïve learning eradicates opinion diversity (the fourth column in Fig. 5). On the other hand, selective exposure fragments groups into subgroups, which become more homogeneous (the third column in Fig. 5) and smaller (the second column in Fig. 5). However, selective exposure leads to no changes in opinion diversity at the global level (the fourth column in Fig. 5), thereby maintaining the quality of collective decisions over time (the fifth column in Fig. 5). It is worth noting that, under strong propensities for selective exposure, the decrease in giant component size $\left|{C}_{1}\right|$ is delayed compared to the changes in other measures. This is because, during the early stage, the whole network remains robust against the modification of local structures due to the high initial connectivity among agents ($k=5$)^81^.

## Discussion

In this study, we have examined the impacts of selective exposure on the quality of collective decisions through an extension of DeGroot’s model^26^ by allowing for endogenous changes in the communication networks of agents. A series of numerical experiments suggest that our models successfully replicate previous findings. The results confirm that naïve learning leads to the convergence of opinions in well-connected groups, and further, collective decisions formed under naïve learning are more accurate than initial individual opinions^30,33,34^. Also, our results are consistent with previous findings, showing that selective exposure leads to the fragmentation of a group into exclusive and homogenous subgroups^53,54,56,57^, each of which converges to a unique consensus^27^. These successful replications of previous findings by our models validate our approach and indicate that the results we present are not in contradiction with the findings of previous studies.

In addition, the present study offers important insights into the understanding of the role selective exposure plays in collective decision-making in comparison to naïve learning, complementing previous findings. The major findings of the current study can be summarized as follows. A group consensus reached through naïve learning is more accurate than initial individual opinions but significantly deviates from the true state of nature, even in the absence of “excessively” influential individuals. Naïve learning progressively improves individual opinions, because the learning process is conceptually equivalent to a collective decision-making process at a small and local scale. However, the rate of improvement decreases, as naïve learning eradicates opinion diversity at both local and global levels at a faster rate than that of improvement. This suggests that the improvement in individual opinion is achieved at the cost of opinion diversity, and that learning from others’ opinions can be beneficial only if those opinions are sufficiently diverse. On the other hand, the diversity-reducing effects of social influence are effectively confined within exclusive subgroups formed by selective exposure, while global-level opinion diversity is well preserved. As a result, the collective decisions made under strong propensities for selective exposure are as accurate as a simple aggregation of independent opinions initially held by individual members. This suggests that selective exposure can function to manage the diversity-reducing effects of social influence in highly interactive communication environments, providing a plausible answer to our motivating question, “How can groups perform well in decision-making *despite* social influence?”.

However, it should be noted that our models examined the effects of selective exposure only in terms of the *accuracy* of collective decisions without considering any potential *costs* associated with coordinating and integrating individuals with diverse or even opposing opinions as well as other negative consequences of structural fragmentation, such as intergroup hostility and conflicts. Perhaps, those costs might exceed the benefits of producing more accurate and unbiased decisions in real-world contexts. Therefore, the current findings should not be interpreted to imply that selective exposure is necessarily superior to other alternative means of reducing disagreement among group members, utilizing local information, or managing the diversity-reducing effects of social influence. Likewise, it is equally undesirable to blindly believe that learning from diverse views or the equivalent would be a “panacea” for a wide range of social and political problems because it leads to poorer and more biased collective decisions than groups could have possibly produced otherwise. This trade-off between the “melting pot” ideal of harmonious consensus and the “salad bowl” concept of preserving diversity deserves further examination. Therefore, future research is required to present more balanced perspectives and theoretical frameworks on opinion dynamics and collective decision-making.

It is important to acknowledge the limitations of our models and discuss the directions of future research to extend the current findings. First, although naïve learning has been widely adopted as a model of opinion dynamics, a problem with its fundamental assumption needs to be carefully considered. Naïve learning is a rational opinion update rule when agents reside in a sparse network, as in such cases, their neighbors’ opinions can be assumed to be effectively independent. However, when the connectivity among neighbors exceeds a certain level, this independence assumption cannot be held. Thus, the failure to recognize and adjust for the excessive connectivity leads to suboptimal social responses and poorer decisions^5^. Nonetheless, the adoption of naïve learning can be defended by assuming that agents lack the cognitive ability to handle the complexity inherent in the densely interconnected networks, they do not have access to the information about the correlation structure among neighbors, and/or they are overly accustomed to situations in which the connectivity among neighbors can be ignored. Alternatively, other formulations of opinion updating can be considered. For example, agents can be assumed to be (Bayesian) rational decision-makers who update their (private) prior opinions based on the observed decisions previously made by others (i.e., sequential decision-making problems). In that case, a high connectivity of network structures leads to the phenomenon known as *information cascades*, where the rational decision-making process is simplified to merely tallying each option chosen by others, and the decision made by a few initial agents takes over the entire group^82–87^. In this scenario, we expect that selective exposure might still function by isolating cascades within the subgroups from which they originate. Also, when selective exposure is viewed as a response to normative social influence by adjusting one’s local structure rather than opinions, its impacts need to be compared to those of alternative ways by which agents avoid disagreement with neighbors without adjusting their own opinions, such as remaining silent^88,89^ or even falsely stating their opinions^27,30^. Buechel et al.,^30^ for instance, consider the possibility that agents intentionally misrepresent their opinions either by conforming or counter-conforming with their neighbors. They show that the misrepresentation of opinions does not necessarily undermine collective accuracy but may even enhance it. According to this framework, “dishonest” agents can uphold their initial opinions despite the normative social influence of their information sources, specifically to the extent that their true opinions differ from their stated opinions. Accordingly, the aggregate of these differences can be seen as the total amount of preserved diversity at the global level. However, the stated opinion of an agent can still influence the opinion formation of other agents who rely on the agent as their information sources. That is, normative social influence is simply “passed along” to other agents instead of being “stopped.” Therefore, it would be interesting to see how the introduction of “dishonest” agents affects the current results.

Second, our models assume that agents initially hold *independent* opinions, the aggregation of which is statistically equal to the true state of nature. However, this independence assumption is not applicable to real-world groups, because individual opinions tend to be correlated with each other even before any social interaction starts. Previous studies show that collective decisions made by a simple aggregation are more accurate when the *average* correlation among agents’ opinions is *negative* than when it is *positive*^90,91^. Although it is impossible for every agent to be mutually contrarian to each other within a group of three or more agents, it is still conceivable that *some* agents’ opinions are *perfectly* negatively correlated with some others, or that *every* agent’s opinion is *moderately* negatively correlated with every other. More specifically, the lower bound of the *average* correlation among *n* agents’ opinions is $\overline{\rho }\ge -1/\left(n-1\right)$ ^90^. Further, when agents’ opinions are maximally negatively correlated, the generalized variance of the joint distribution of *n* opinions (i.e., multivariate dispersion) is accordingly maximized, maintaining opinion diversity within groups. Therefore, collective accuracy can be significantly improved by incentivizing individuals to be in the minority^6^. Further, whereas our model assumes no changes in opinions *after* selective exposure, the reinforcement model^55,92^—a framework proposed to account for the personalization-polarization hypothesis^93^—predicts that individuals tend to develop more extreme views after interacting with like-minded others. If that is the case, selective exposure widens the gaps between opinions that are initially leaning toward different ends, and the correlation between them grows in the negative direction, *enhancing* the quality of collective decisions rather than merely maintaining it. Perhaps, a *positive* correlation among individual opinions is more common to real-world groups, even though their effects on collective accuracy become negligible as group size increases^94^. In particular, the *external* factors of correlated opinions (i.e., “induced homophily” as opposed to preference-based “choice homophily”^95^), such as spatial constraints^39^ and the reliance on common information sources^45^, should be duly incorporated into our models. One possible way is to include those factors as a set of pseudo-agents with no incoming edges but with a disproportionately large number of out-going edges, forming a bipartite network in which one mode is composed of the pseudo-agents and the other consists of the “ordinary” agents. These relatively straightforward extensions could help clarify how these additional factors affect the causal path from selective exposure to the quality of collective decisions through group structure and opinion diversity.

### Supplementary Information

Supplementary Information 1.

Supplementary Information 2.

Supplementary Information 3.

Supplementary Information 4.

Supplementary Information 5.

Supplementary Information 6.

Supplementary Information 7.

Supplementary Information 8.

## Data Availability

The datasets generated by numerical experiments and Python scripts used for simulations are available from the corresponding author on reasonable request.
