# Supplementary material for: The functional aspects of selective exposure for collective decision-making under social influence
Source: Sci Rep. 2024 Mar 17;14:6412. doi: 10.1038/s41598-024-56868-8 (PMC10944847; doi:10.1038/s41598-024-56868-8)
Supplement: Supplementary file 1 — Supplementary Information 1. [file 41598_2024_56868_MOESM1_ESM.docx]

**The Functional Aspects of Selective Exposure for Collective Decision-making under Social Influence: Supplementary Materials**

**Poong Oh^1*^, Jia Wang Peh^2^, and Andrew Schauf^3^**

^1^ Wee Kim Wee School of Communication and Information, Nanyang Technological University, 639798, Singapore

^2^ Information Systems Technology and Design Pillar, Singapore University of Technology and Design, 487372, Singapore

^3^ Department of Physics, National University of Singapore, 119077, Singapore

^*^[poongoh@ntu.edu.sg](mailto:poongoh@ntu.edu.sg)

These Supplementary Materials present the results of numerical experiments with additional settings to establish the robustness of the results reported in the main article, *The Functional Aspects of Selective Exposure for Collective Decision-making under Social Influence*. Section S1 reviews the statistical behavior of groups assumed in classical models of collective decision-making—Condorcet’s theorem^1^, Galton’s experiment^2^, and DeGroot’s model^3^, based on which the current models are developed. Section S2 briefly describes our models and extended simulation settings, Section S3 then presents the results, and Section S4 summarizes the main findings.

# S1. Models of Collective Decision-making

## S1.1. Collective Decisions by Independent Individuals

Condorcet’s (jury) theorem^1^ was introduced in 1785 as a theoretical basis for modern democracy during the Age of Enlightenment. The theorem assumes that there are two decisional alternatives (e.g., ‘agree’ or ‘disagree’; ‘guilty’ or ‘not guilty’), one of which is correct in an objective way but is unknown to individuals. Individuals are assumed to choose the correct decision with a probability of $p>.5$ (namely, *individual competence*). In that case, individuals do not have to be always correct. Instead, it suffices that they are just rational enough to be slightly more likely to make the correct decision than the wrong decision. This assumption can be relaxed, allowing the mean individual competence to be greater than .5 (i.e., $\bar{p}>.5$)^4^. Lastly, all decisions are assumed to be made independently and then aggregated into the group’s decision by the majority rule. Under these assumptions, the probability that a group arrives at the correct decision (i.e., *collective performance*) is always greater than individual competence $p$. More importantly, this probability increases monotonically and approaches 1 in the limit as group size increases.

To illustrate the statistical behavior of groups described in the theorem, consider that individual decision $X$ is an *i*.*i*.*d*. Bernoulli random variable with a success probability or individual competence $p$:

$$P\left( X=x \right)=\left\{ \begin{matrix} p & if x=1 & (\text{True}) \\ 1-p & if x=0 & (\text{False}) \end{matrix} \right.$$

Then, the number of correct decisions in a group of $n$ individuals follows a binomial distribution, $Y=\sum_{i}^{n} X_{i} \sim Bin\left( n,p \right)$. Further, the probability of the group reaching the correct decision by the majority rule, or put differently, the probability that more than half of the individuals in the group make the correct decision $P_{G}$, is defined as the sum of probability mass for $Y>n/2$:

$$P_{G}\equiv P\left( Y>\frac{n}{2} \right)=\sum_{y>n/2}^{n} \left( \begin{matrix} n \\ y \end{matrix} \right)p^{y}\left( 1-p \right)^{n-y}.$$

When individual competence $p$ is greater than .5, the mean $np$ of the binomial distribution always exceeds $n/2$. Also, the sum of the probability mass $P\left( Y>n/2 \right)$ is always greater than $p$, because the distribution is skewed to the left, having a negative skewness $\frac{1-2p}{\sqrt{np(1-p)}}$. Further, as $n$ increases, variability around the mean decreases, and the probability that the group is wrong [i.e., $P\left( Y\leq\frac{n}{2} \right)$] converges to 0 by the central limit theorem, implying that sufficiently large groups will almost always reach the correct decision. However, it is important to note that the variability around the mean $\left| \sum_{i}^{n} X_{i}-np \right|$ is inversely proportional to the square root of group size $\sqrt{n}$. This implies that the marginal improvement of collective performance diminishes as the group size increases^5^.

Whereas the individuals in Condorcet’s theorem are to make a binary decision, individuals in Galton’s experiment^2^ were asked to make a continuous decision, specifically, to guess the weight of a live ox. Galton observed that the “middlemost” estimate of valid guesses (i.e., “valid” in the sense of being uninfluenced by others’ guesses) was accurate within a 1% margin of the ox’s true weight, and the mean estimate was even more accurate^6^. Based on this observation, Galton argued that the simple aggregation of independent judgments can produce a surprisingly accurate collective outcome because the errors in individual judgments are normally distributed, and therefore cancel each other out when aggregated.

To illustrate this point, suppose that individual opinions $X$ follow a normal distribution with mean $\mu$ and variance $\sigma^{2}$, where $\mu$ is assumed to be the true value. Then, consider individual errors as the squared deviations of individual decisions from the true value: $\left( X_{i}-\mu\right)^{2}$. Because $\left( X_{i}-\mu\right)/\sigma$ follows the standard normal distribution, $\left( X-\mu\right)^{2}/\sigma^{2}$ follows the chi-squared distribution with degree of freedom 1, which is equivalent to a gamma distribution with shape parameter 1/2 and scale parameter 2:

$$\frac{\left( X_{i}-\mu\right)^{2}}{\sigma^{2}} \sim\chi^{2}\left( 1 \right)=\Gamma\left( \frac{1}{2}, 2 \right).$$

Therefore, individual errors follow a gamma distribution with shape parameter 1/2 and scale parameter 2$\sigma^{2}$:

| $\left( X_{i}-\mu\right)^{2} \sim\sigma^{2}\Gamma\left( \frac{1}{2}, 2 \right)=\Gamma\left( \frac{1}{2}, 2\sigma^{2} \right).$ | (1) |
| --- | --- |

Similarly, consider a collective decision as the group mean of $n$ individual opinions $\bar{X}=\frac{1}{n}\sum_{i}^{n} X_{i}$ and the collective error as the squared deviation from the true value: $\left( \bar{X}-\mu\right)^{2}$. By the central limit theorem, $\left( \bar{X}-\mu\right)$ follows the normal distribution with mean $\mu$ and variance $\sigma^{2}/n$, and $\left( \bar{X}-\mu\right)^{2}/\left( \sigma^{2}/n \right)$ follows the chi-squared distribution with degree of freedom 1, which is equivalent to a gamma distribution with shape parameter 1/2 and scale parameter 2:

$$\frac{\left( \bar{X}-\mu\right)^{2}}{\sigma^{2}/n} \sim\chi^{2}\left( 1 \right)=\Gamma\left( \frac{1}{2}, 2 \right).$$

Therefore, collective errors follow a gamma distribution with shape parameter 1/2 and scale parameter 2$\sigma^{2}/n$:

| $\left( \bar{X}-\mu\right)^{2} \sim\frac{\sigma^{2}}{n}\Gamma\left( \frac{1}{2}, 2 \right)=\Gamma\left( \frac{1}{2},\frac{2\sigma^{2}}{n} \right).$ | (2) |
| --- | --- |

The two gamma distributions above (Eqs. 1 and 2) share a common shape parameter 1/2, but the scale parameter for individual errors is *n* times larger than that of collective errors. This means that the collective opinion $\bar{X}$ is statistically *n* times closer to the true value than individual opinions $X$ are. Moreover, as *n* increases, collective error $\left( \bar{X}-\mu\right)^{2}$ converges to zero, suggesting that larger groups make more accurate decisions. However, collective error $\left( \bar{X}-\mu\right)^{2}$ is inversely proportional to group size *n*, suggesting that the marginal improvement of collective performance diminishes as the group size increases^5^ as in Condorcet’s theorem.

Even though they assume different types of opinions (i.e., binary or continuous opinions) and adopt different methods for aggregating individual decisions into collective decisions (i.e., the majority rule, the median or mean aggregation), both Condorcet and Galton reach the same conclusion: Groups outperform individuals in decision-making, and the quality of collective decisions monotonically increases as a function of group size. However, both hold the unrealistic assumption that individuals in groups make their decisions *independently* without influencing or being influenced by others.

## S1.2. Collective Decisions under Social Influence

Perfect independence of opinions is hardly expected in real-world groups because social influence between group members is inevitable in highly interactive communication environments. To formulate a model of opinion dynamics under social influence, DeGroot’s theorem^3^ allows individuals to naïvely update their opinions by repeatedly adopting the average opinion in their information sources, in a similar way that real-life participants have been observed to reduce the disagreement with their peers in various social psychological experiments^7^. The theorem then identifies the conditions under which a group of individuals with diverse opinions reach a consensus.

The theorem formulates the update of individual opinions as a Markov process. An individual opinion $x_{i}$ at a discrete time point $t\in\left\{ 1, 2, 3, \ldots\right\}$ is determined as the weighted mean of others’ opinions in the previous round at $t-1$:

$$x_{i}\left( t \right)=w_{i1}x_{1}\left( t-1 \right)+w_{i2}x_{2}\left( t-1 \right)+\ldots+w_{in}x_{n}\left( t-1 \right),$$

where $w_{ij}$ denotes the relative importance of *j*’s opinion with respect to the update of *i*’s opinion ($\sum_{j} w_{ij}=1$ and ${0\leq w}_{ij}\leq1$). This can be rewritten in matrix notation as follows:

$\mathbf{x}\left( t \right)=\mathbf{Wx}\left( t-1 \right)$ or $\mathbf{x}\left( t \right)=\mathbf{W}^{t}\mathbf{x}\left( 0 \right)$, where $\mathbf{W}\boldsymbol{=}\left[ w_{ij} \right].$

Since **W** is a row-stochastic matrix (i.e., $\sum_{j} w_{ij}=1$ for all $i$), $\mathbf{x}\left( t \right)$ converges to a steady-state distribution $\mathbf{x}^{\boldsymbol{*}}$ in the limit:

$$\mathbf{x}^{\boldsymbol{*}}=\lim_{t\to\infty} \mathbf{x}\left( t \right)=\lim_{t\to\infty} \mathbf{W}^{t}\mathbf{x}\left( 0 \right)=\mathbf{v}_{1}\mathbf{x}\left( 0 \right),$$

where $\mathbf{v}_{1}$ is the eigenvector corresponding to the leading eigenvalue of **W**, which is equal to 1.

The theorem shows that all the entries of $\mathbf{x}^{\boldsymbol{*}}$ become equal under two conditions: First, the Markov process is capable of transitioning from any state to any other state with a non-zero probability, having no isolated subset of states (i.e., irreducibility). Second, the process does not exhibit a regular, repeating pattern in its transition (i.e., aperiodicity). In the context of collective decision-making, these two conditions can be interpreted as follows: So far as the communication network of group members, where individuals are connected by directed weighted edges $w_{ij}$, remains strongly connected (i.e., every member is connected to everyone else by at least one directed path), every member will eventually adopt a common opinion, and so the group will reach a consensus.

Notice that the group consensus is given by the weighted mean of initial individual opinions $\mathbf{v}_{1}\mathbf{x}\left( 0 \right)$, which is not equal to the simple mean aggregation of initial opinions $\frac{1}{n}\mathbf{1x}\left( 0 \right)$. Because $\mathbf{v}_{1}$ is equivalent to the eigenvector centralities of individual agents in the network^8^, the group consensus reached by a strongly connected group is not necessarily the “middlemost” opinion that evenly reflects the diverse opinions held by individual members at the beginning. Instead, it is determined—or at least highly influenced—by a few individuals who occupy the most central positions in the group.

# S2. Agent-based Models

In the main article, we consider four agent-based models (*Condorcet*, *Galton*, *Bimodal*, and *Exponential* *Models*), each of which represents a unique collective decision-making setting characterized by its own initial opinion distribution, aggregation method of forming collective decisions, and the operationalization of individual and collective performances.

## S2.1. Common Settings

All the four models consider groups of *n* agents. Each agent starts with its opinion $x_{i}$ that is an *i*.*i*.*d*. random variable that follows the probability distribution specified in each model. This ensures that individual opinions are independent from one another at the beginning of each simulation. Also, each agent is connected to *k* other agents that serve as its information sources. The connections among agents are directed, not necessarily reciprocal, and independent of their initial opinions, ensuring that agents are initially exposed to diverse opinions. The communication network of agents is denoted by *G,* and its adjacency matrix $A_{ij}$ represents the directions of influence from agents *i* to *j*. The set of agent *i*’s information sources, or *information pool*, is denoted by $G\left( i \right)=\left\{ j \right| a_{ij}=1\}$.

In each round, agents attempt to minimize discrepancies with their information sources in a random order. Discrepancy is measured as the sum of differences between an agent’s opinion and those of its information sources $\sum_{j\in G\left( i \right)} \left| x_{i}-x_{j} \right|$ (i.e., the sum of absolute deviations). We consider two ways of reducing discrepancy, the first of which is *naïve learning*, by which an agent simply adopts the opinion $x_{i}^{'}$ such that it minimizes the discrepancies with its information sources:

$$x_{i}^{'}=\arg\min\sum_{j\in G\left( i \right)} \left| x_{i}-x_{j} \right|.$$

Alternatively, agents can reduce discrepancy via *selective exposure*, by which an agent removes its connection to the information source whose opinion differs most from its own and adds a new connection to a randomly selected agent whose opinion is more congruent with its own than the removed one. In each round, each agent chooses either selective exposure with a probability of $\beta$ or naïve learning with its complement of $1-\beta$. A set of 51 evenly spaced values between 0 and 1 are used to quantify the parameter describing agents’ propensity for selective exposure: $\beta\in\{.00,.02,.04.,\ldots,1.00\}$. For each value of $\beta$, a total of 30,000 simulations are performed. Each simulation is terminated when no changes are observed either in connections or in opinions.

## S2.2. Model Specific Settings

The *Condorcet Model* assumes that initial individual opinion $x_{i}\in\left\{ True, False \right\}$ for $i=1, 2, 3, \ldots, n$, is an *i*.*i*.*d* Bernoulli random variable. Individual opinions at a steady state are considered as individual decisions, and the decision chosen by more group members between the two decisions is considered as the group’s collective decisions (i.e., the majority rule).

The *Galton Model* assumes that initial individual opinion $x_{i}$ is an *i*.*i*.*d* standard normal random variable $x_{i} \sim N(\mu=0, \sigma^{2}=1)$, where the true state is $\mu=0$, against which individual and collective decisions are to be compared. The collective decision $\bar{x}$ is determined as the mean of individual opinions at steady state (i.e., mean aggregation).

The *Bimodal Model* assumes that initial individual opinions $x_{i}$ follow a mixture of two normal distributions with different means $\mu_{j}\in\left\{ -2, 2 \right\}$ and a common variance $\sigma_{j}^{2}=1$ such that the distribution has two peaks, representing polarized opinion distributions. The collective decision $\bar{x}$ is determined as the mean of individual opinions at steady states (i.e., mean aggregation).

The *Exponential Model* assumes that initial individual opinions $x_{i}$ follow an exponential distribution with rate parameter $\lambda=1$, representing skewed opinion distributions. The collective decisions are determined as the *median* of individual opinions (i.e., median aggregation) to minimize the influences of outliers. Accordingly, the true state is set as the median of the distribution, $\tilde{\mu}=\ln2=0.6931$.

## S2.3. Measures

In the *Condorcet Model*, *individual* and *collective performances* are measured as follows: Individual performance $p_{i}$ is measured by the proportion of agents whose final decisions are *True*, and collective performance $P_{G}$ by the proportion of groups whose majority decide *True* at steady states. To measure individual and collective performances in the other models, we first operationalize “Good Decisions” as those that fall within two standard errors from the true state $\left\{ \bar{x}| \left| \bar{x}-\mu_{\bar{x}} \right|<2\sigma_{\bar{x}} \right\}$, where the standard error is $\sigma_{\bar{x}}=\sigma/\sqrt{n}$, and $\mu$ is the true state ($\tilde{\mu}$ for the Exponential Model) specified by a model. Then, both individual performance $p_{i}$ and collective performance $P_{G}$ are defined as the proportions of “Good Decisions” made at steady states.

In addition, we measure *opinion diversity* $\sigma_{x}$, the size of *giant component* $\left| C_{1} \right|$, and *modularity* $Q$ at steady states to examine the impacts of local dynamics on the global structure. First, opinion diversity $\sigma_{x}$ is measured by the standard deviation of individual opinions at steady states. Second, the size of giant components $\left| C_{1} \right|$ is measured by the proportion of nodes that belong to the largest strongly connected component of a communication network $G$, within which every node is reachable from every other node through one or more directed paths. Further, modularity $Q$ is measured as

$$Q=\sum_{d} \left[ \frac{L_{d}}{m}-\left( \frac{k_{d}^{\mathrm{in}}k_{d}^{\mathrm{out}}}{2m} \right)^{2} \right],$$

where $d$ represents the dichotomized categories of individual opinions (i.e., *True* or *False*; *above* or *below* the true value), $m$ is the total number of edges, $L_{d}$ is the total number of edges within category $d$, and $k_{d}^{\mathrm{in}}$ and $k_{d}^{\mathrm{out}}$ are the sums of in-degrees and out-degrees of the nodes in category $d$.^9^ Low values of $\left| C_{1} \right|$ and high values of $Q$ indicate the presence of echo chambers in groups.

## S2.4. Additional Settings

We reexamined the four models with different settings to establish the robustness of the results reported in the main article. Those settings include different group sizes ($n=200$ and $n=400$ for the *Galton* and *Bimodal Models*; $n=201$ and $n=401$ for *Condorcet* and *Exponential Models*), numbers of information sources ($k=3$ and $k=9$), and initial network structure (i.e., directed regular networks and scale-free networks).

A directed *k*-regular network refers to a network where every node has an equal number of outgoing edges $k^{out}$ and incoming edges $k^{in}$, which means that every agent in the group not only is influenced by an equal number of other agents but also influences an equal number of other agents. Because the connections among agents are not necessarily reciprocal, the set of agents who influence an agent may not be the same as the set of agents who are influenced by the agent. We employ an algorithm to generate a directed *k*-regular network: In each round of the network generation process, each agent *i* randomly selects another agent *j* who has less than *k* outgoing edges in a random order, and such a round is repeated *k* times. Although it may not be as efficient as previous algorithms, this simple algorithm generates networks with no self-loop or parallel edges (unlike the configuration model^10^) and does not require the product of the number of nodes and the number of outgoing edges per node $nk^{out}$ to be even (unlike the algorithm for regular graphs^11^). Most importantly, every node has the exact same eigenvector centrality. Therefore, a directed *k*-regular network represents a perfectly *decentralized* group where every agent exerts an equal amount of influence on the group’s collective decisions according to DeGroot’s theorem^3^.

On the other hand, directed scale-free networks are generated according to a preferential attachment mechanism^12^. Specifically, each agent *i* selects its information source *j* with a probability proportional to the source’s out-degree:

$$P \left( i\leftarrow j \right)\propto\frac{k_{j}^{out}+1}{\sum_{j} (k_{j}^{out}+1)}.$$

In each round, each agent selects an information source and forms an incoming edge in a random order, which is then repeated for *k* rounds.

Figure S1a visualizes the out-degree distributions averaged over 30,000 networks of each type generated for *Galton Model* ($n=100$). The out-degree of every agent in the regular networks (the red vertical bar) is constant as $k^{out}=k^{in}=5$. The out-degree distribution of the random networks (the green histogram) approximately follows a binominal distribution $k^{out}\sim Bin\left( n=99, p=.05 \right)$. The out-degree distribution of the scale-free networks (the blue histogram) approximately follows a power-law with an exponent parameter $\alpha=-3.277$, displaying a long and thick tail.

Figure S1b presents the cumulative eigenvector centralities of the three different network types, which are equivalent to the weights of initial individual opinions on the group consensus reached by the Markov process described in DeGroot’s theorem. In regular networks, cumulative eigenvector centrality increases linearly (the red line), suggesting that every agent exerts exactly an equal amount of influence on the group consensus. Therefore, the group consensus is merely the unweighted mean of initial individual opinions, reflecting every agent’s opinion evenly. On the other hand, cumulative eigenvector centralities are shown as upward convex curves with respect to rank in eigenvector centrality in random networks (the green curve) and scale-free networks (the blue curve). This suggests that some agents who occupy the central positions in the group exert disproportionate influence on group consensus. In a scale-free network, for example, the total influence of the top 16 central agents amounts to 50% of influence upon the group consensus. Furthermore, the top 50 agents exert 90% of total influence, whereas the other 50 agents contribute only 10%. This means that the consensus reached by a group with a highly centralized structure is mostly determined by just a few central individuals and tends to ignore the diverse opinions of peripheral others.


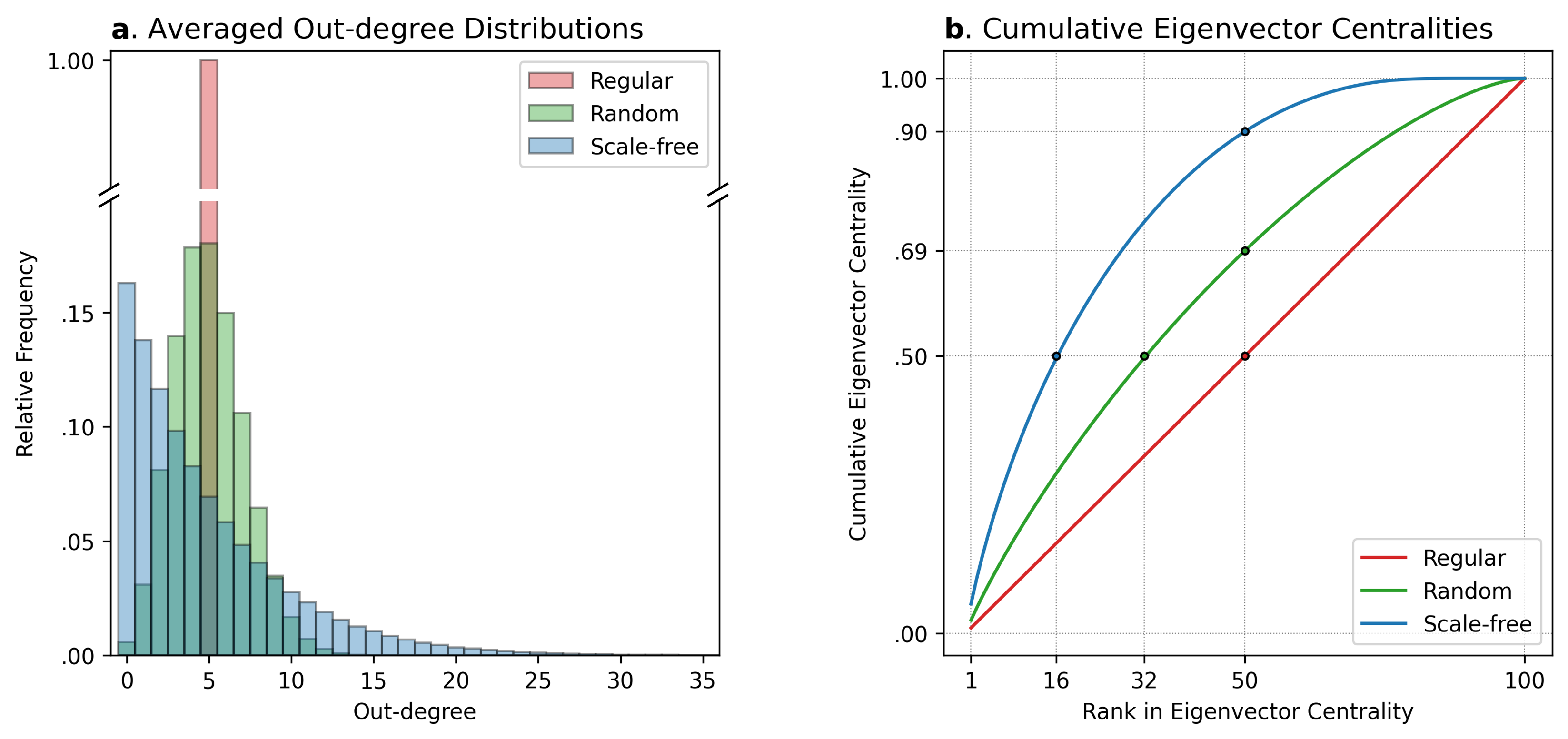


*Figure S1*. Averaged out-degree distributions (**a**) and cumulative eigenvector centralities (**b**) of 30,000 regular (red), 30,000 random (green), and 30,000 scale-free (blue) networks, where the number of agents is $n=100$.

# S3. Results

The results presented in the main article consistently suggest that *naïve learning* eradicates opinion diversity within groups, and thereby significantly undermines the quality of collective decisions across all the models. On the other hand, *selective exposure* preserves opinion diversity and maintains the quality of collective decisions at a level as high as that achieved under perfect independence. Whereas the effects of selective exposure observed in the main study are replicated here over a range of different settings, the effects of naïve learning are found to vary depending on the size of information pool and group structure.

## S3.1. The Effects of Group Size

Both Condorcet’s theorem and Galton’s experiment suggest that collective performance monotonically increases as group size increases. To examine the impacts of group size, numerical experiments are performed with different group sizes ($n=200$ and $n=400$ for the *Galton* and *Bimodal Models*; $n=201$ and $n=401$ for the *Condorcet* and *Exponential Models*) from those used in the main article. The results are summarized in Figures S2 and S3.

Although the overall patterns remain nearly identical to those reported in the main article, subtle differences are found in individual and collective performances. In the *Condorcet Model*, specifically, collective performance under the maximal propensity for selective exposure ($\beta=1$) increases from .844 ($n=101$) to .923 ($n=201$) and .978 ($n=401$). This is to be expected from Condorcet’s theorem. Also, collective performance under the maximal propensity for naïve learning ($\beta=0$) increases from .749 ($n=101$) to .827 ($n=201$) and .907 ($n=401$). Because a larger group slows down the reduction of opinion diversity at the global level, the improvement in individual opinions continues over a longer period.


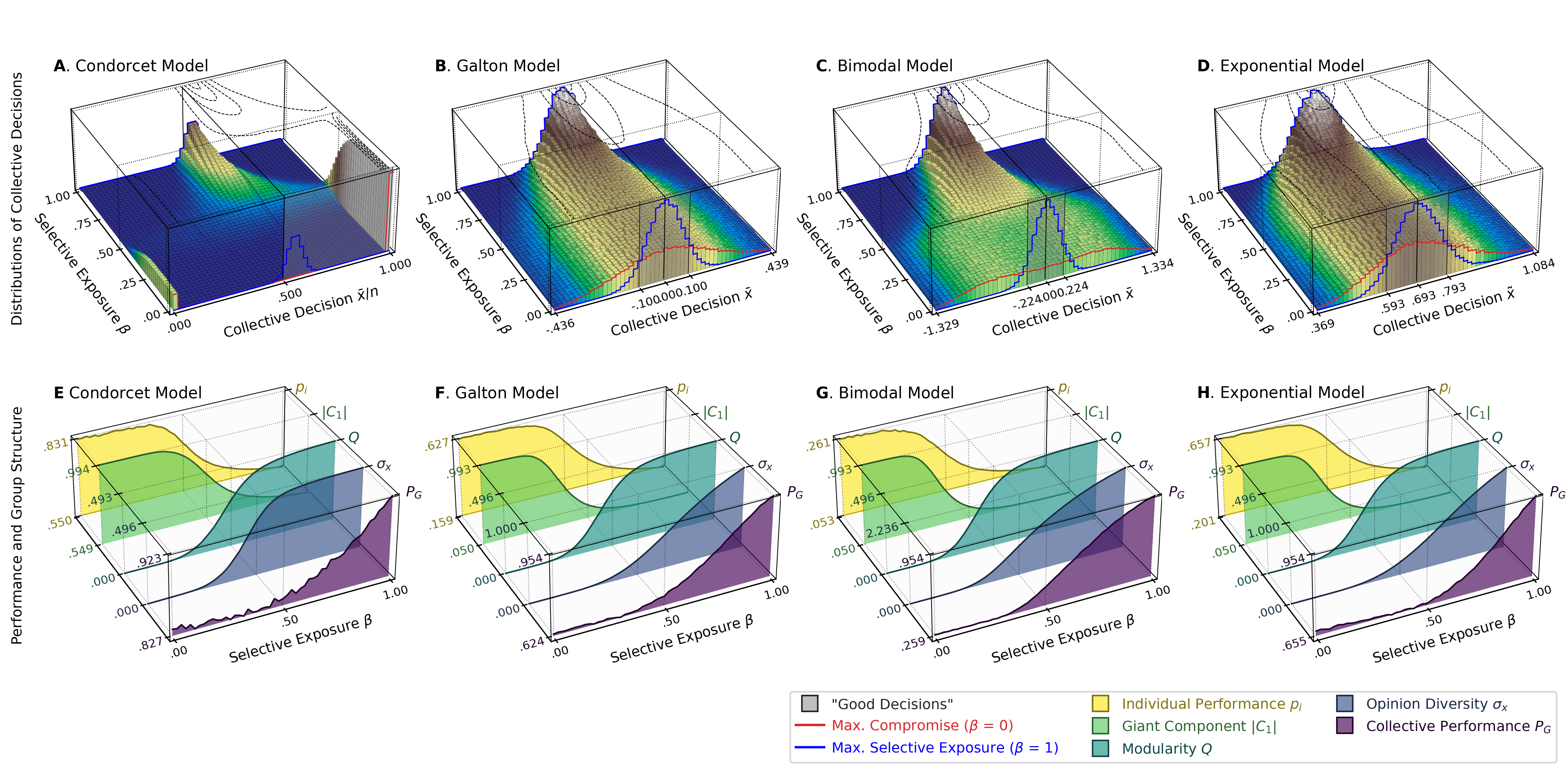


*Figure S2*. The distributions of collective decisions (**a** through **d**) and metrics describing performance and group structures (**e** through **h**), where group size is set as $n=200$ for the *Galton* and *Bimodal Models* and $n=201$ for the *Condorcet* and *Exponential Models*.


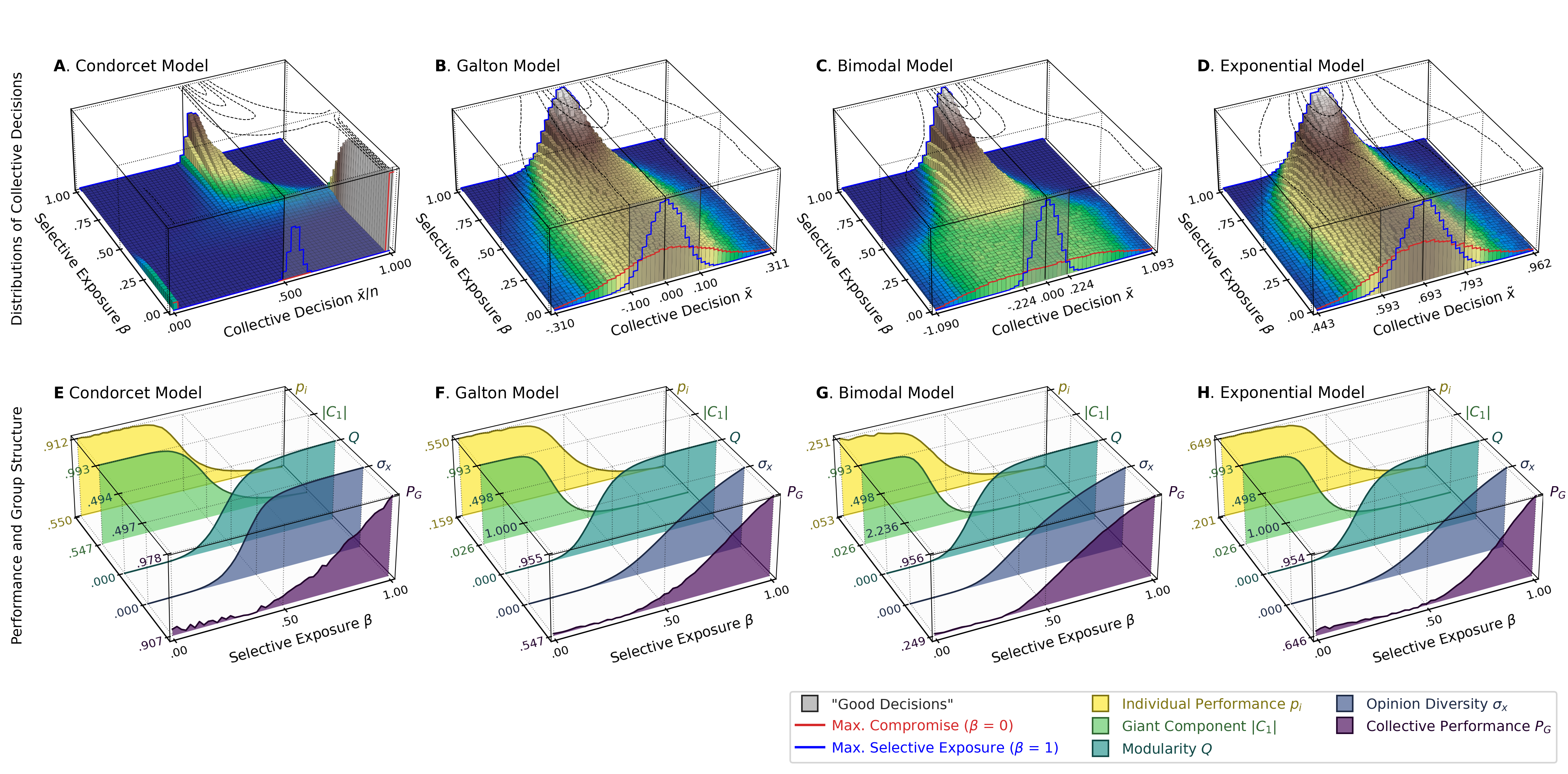


*Figure S3*. The effects of selective exposure on the distributions of collective decisions (**a** through **d**) and metrics describing performance and group structures (**e** through **h**), where group size is $n=400$ for the *Galton* and *Bimodal Models* and $n=401$ for the *Condorcet* and *Exponential Models*.

On the other hand, collective performance under the maximal propensity for selective exposure ($\beta=1$) remains unchanged in the other three models. This is because the criterion defining “Good Decisions” is determined by the standard errors $\sigma_{\bar{x}}$, which already accounts for group size *n*. Interestingly, however, significant decreases in collective performances under the maximal propensity for naïve learning ($\beta=0$) are consistently observed across all three models (e.g., from .712 to .624 and .547 in the *Galton Model*). This suggests that collective performance under naïve learning becomes worse *relative* to collective performance under selective exposure. From this, it can be inferred that the gap between the qualities of collective decisions formed under selective exposure and naïve learning increases as groups become larger, suggesting that the marginal improvement in collective decisions diminishes as group size grows.

## S3.2. The Effects of the Size of Information Pool

Next, we examine the effects of the number of information sources per agent ($k=3$ and $k=9$), who serve as an agent’s information sources.

When $k=3$ (Figure S4), groups are more easily fragmented by selective exposure. Therefore, opinion diversity is better preserved, and the quality of collective decisions are better maintained too even at a lower level of selective exposure across all the models. Individual performance is still improved under naïve learning ($\beta=0$) but not as much as when $k=5$ (i.e., the results shown in the main article). This is to be expected, because individual agents update their opinions relying on a smaller number of information sources.


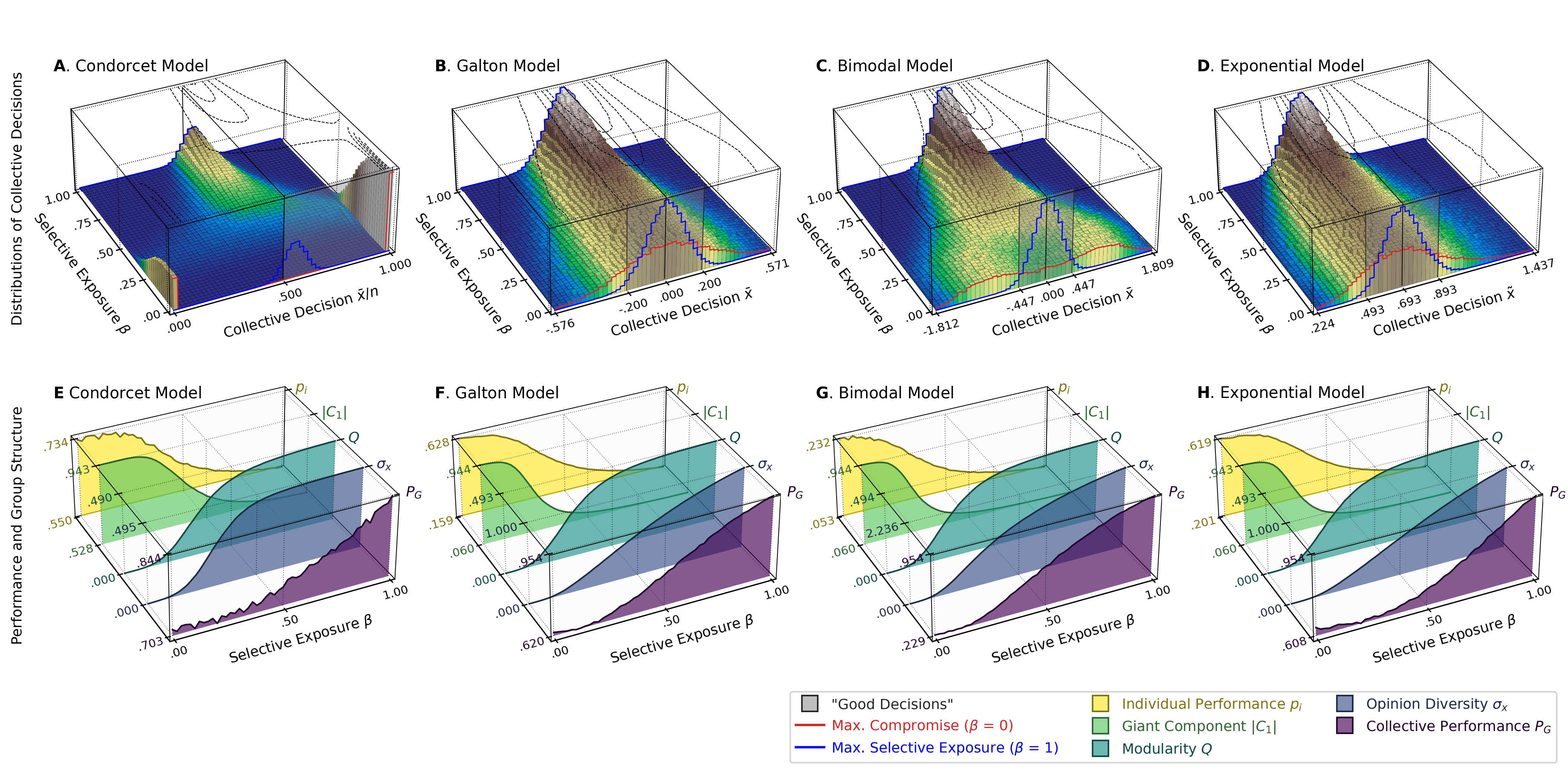


*Figure S4*. The distributions of collective decisions (**a** through **d**) and metrics describing performance and group structures (**e** through **h**), where the number of information sources is set as $k=3$.

When $k=9$ (Figure S5), symmetric results are obtained: Groups are unlikely to be fragmented and more likely to remain connected even at a higher level of selective exposure, which significantly inhibits the functions of selective exposure in preserving opinion diversity and maintaining the quality of collective decisions across all the models. However, individual performance is much improved under naïve learning ($\beta=0$), because individual agents rely on a larger number of information sources as they update their opinions. Nevertheless, this improvement in individual performance does not necessarily produce an overall improvement in collective performance, suggesting that selective exposure still pays off more for collective decision-making.


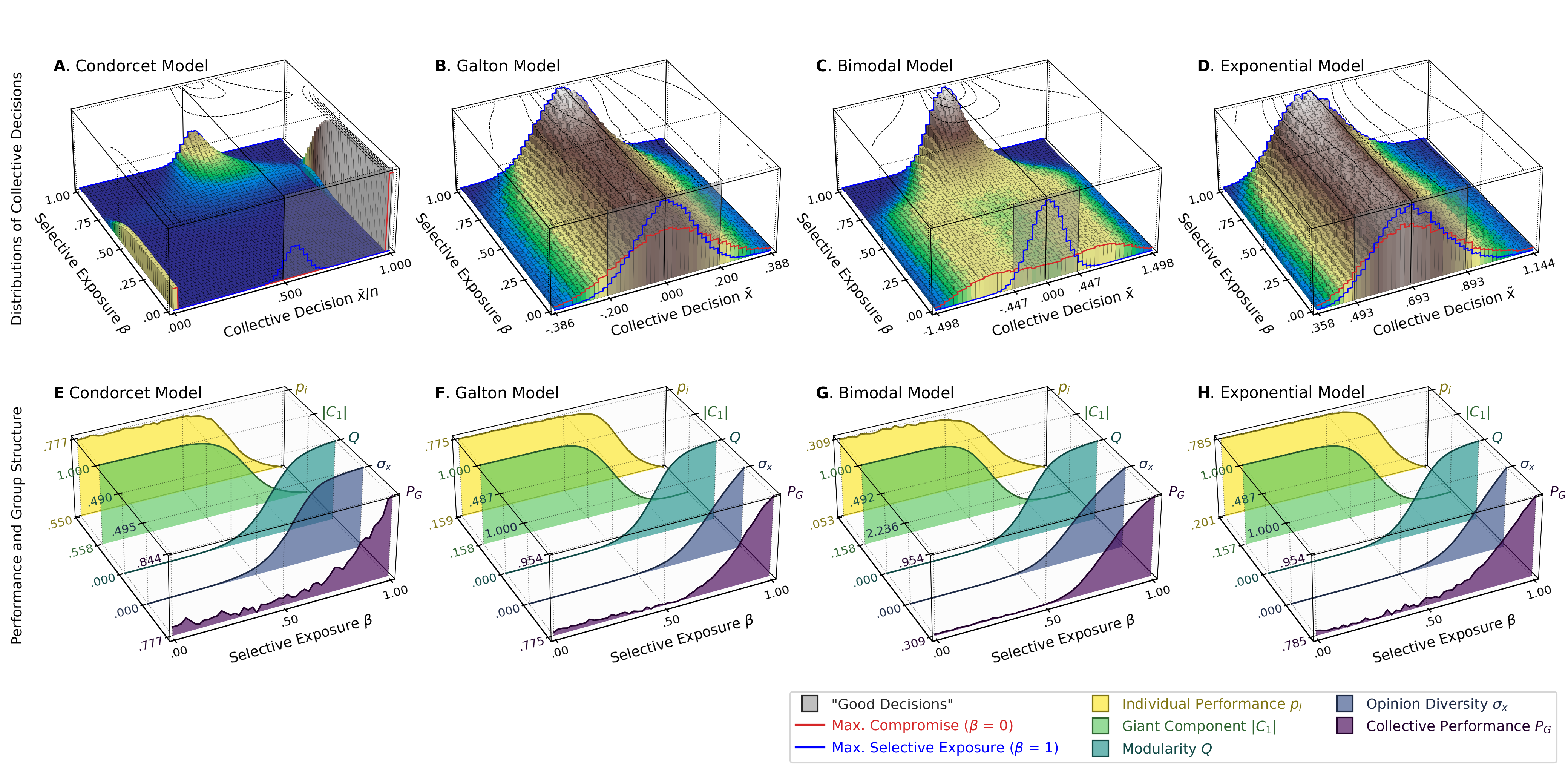


*Figure S5*. The distributions of collective decisions (**a** through **d**) and metrics describing performance and group structures (**e** through **h**), where the number of information sources is set as $k=9$.

## S3.3. The Effects of Initial Group Structure

Finally, we examine our models with different initial group structures, including directed regular networks and scale-free networks. However, it is expected that initial group structures should have little to no impact on collective decision-making under strong propensities for selective exposure, because agents will collectively modify group structures such that the tendency for homophily is maximized. On the other hand, initial group structures are expected to exert a stronger influence upon outcomes under strong propensities for naïve learning.


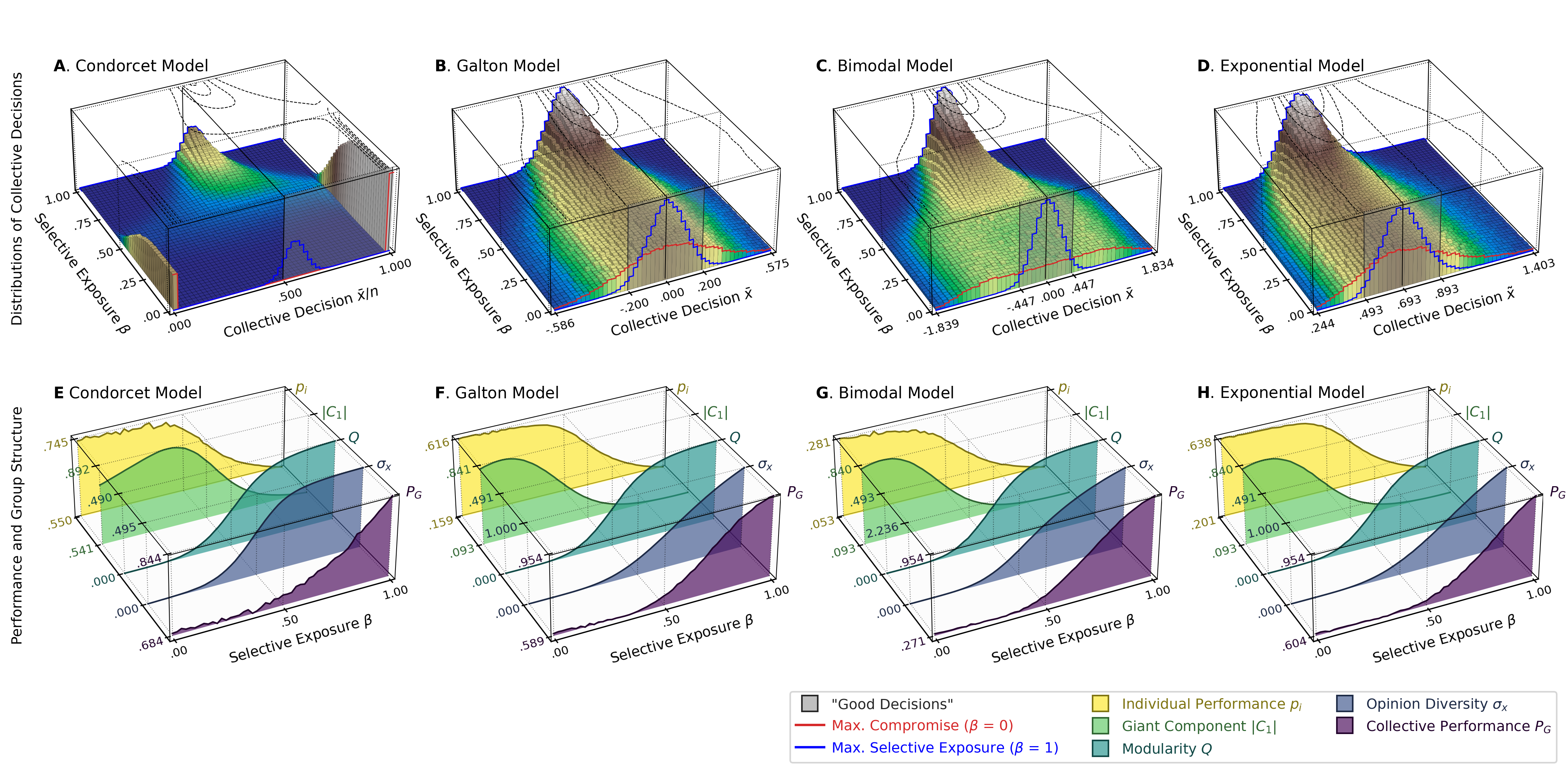


*Figure S6*. The distributions of collective decisions (**a** through **d**) and metrics describing performance and group structures (**e** through **h**), where initial group structures are *scale-free networks*.

When the initial group structures are scale-free networks (Figure S6), individual and collective performances and final group structures are nearly identical to those observed for initial random networks (i.e., the results reported in the main article) under the maximal propensity for selective exposure ($\beta=1$), as expected. In contrast, more significant differences are observed under the maximal propensity for naïve learning ($\beta=0$). First, the distributions of collective decisions are more dispersed (Figure S6a through S6d), and accordingly, the quality of collective decisions is further lowered. This is because collective decisions are subject to the disproportionate influence of a few central agents in groups. More importantly, individual decisions are not improved by naïve learning as much as they are under other settings because different agents likely rely on the same information sources (i.e., the few same central agents) to update their opinions, which reduces opinion diversity within their individual information pools.

Perhaps the most interesting results are obtained when initial group structures are directed regular networks, where the eigenvector centrality of every agent is equal, and thus every agent exerts an equal amount of influence upon collective decision-making. Unlike all the other simulation settings examined here and in the main article, across all four models, the maximal propensity for naïve learning ($\beta=0$) produces collective decisions just as good as those made under the maximal propensity for selective exposure ($\beta=1$) and under the condition of perfect independence. This is because the group consensus reached in a directed regular network is given by the unweighted mean of initial individual opinions. More interestingly, the relationship between selective exposure propensity and collective performance is found to be V-shaped, showing that the worst collective performances are observed over a range of $\beta$ values between .5 and .6, where the two strategies are well mixed. This suggests that when it comes to collective decision-making, these mixed strategies neither enjoy the benefits of selective exposure nor those of naïve learning.


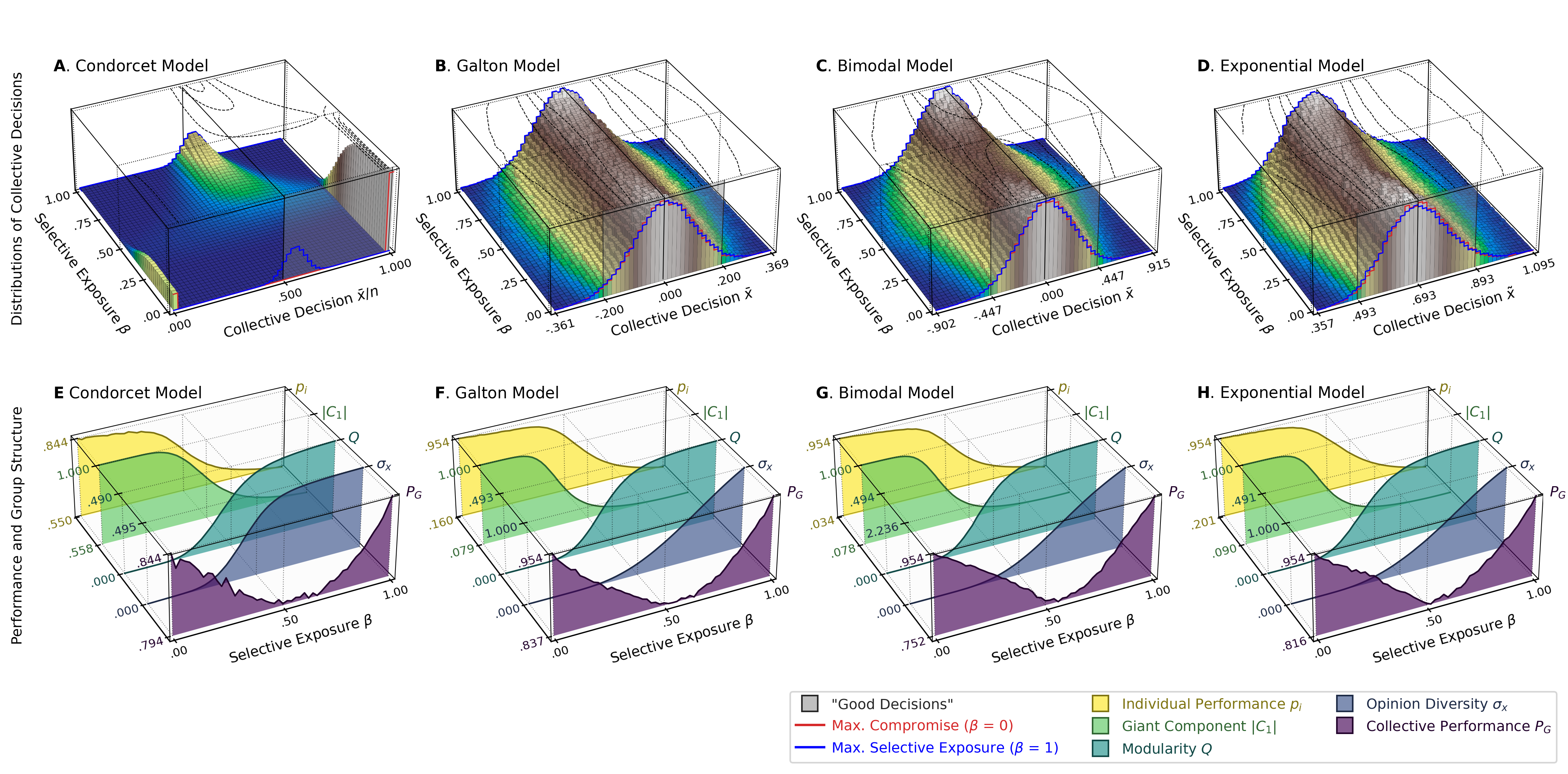


*Figure S7*. The distributions of collective decisions (**a** through **d**) and metrics describing performance and group structures (**e** through **h**), where initial group structures are *directed regular networks*.

# S4. Summary

The findings reported in the main article are successfully replicated in the extended numerical experiments performed here over a wider range of different settings, confirming the robustness of our findings: Selective exposure preserves opinion diversity within groups and thereby maintains the quality of collective decisions at levels just as high as those achieved under the conditions of perfect independence.

In addition, these extended numerical experiments allow us to identify the conditions under which naïve learning can also act to maintain the quality of collective decisions. First, when each agent has access to a large pool of information, it can improve its individual decisions as well as collective decisions, which can be inferred based on the results in Section S2.2. Ideally, if every agent can be exposed to and has a chance to learn from everyone else’s opinions (i.e., $k=n-1$), collective decisions under naïve learning should be as good as those made under selective exposure or in the absence of social influence. Another condition is that the structures of groups should be perfectly *decentralized*, and so all group members must exert an equal amount of influence on the group consensus. There must be neither “leaders” nor “followers” for this to hold true. Perhaps these conditions might be satisfied in a hypothetical democratic utopia that is described by the idealized concept of the “public sphere”^13^ but which has never existed in reality^14,15^.

# References

1. Condorcet, N. D. *Essai sur l’application de l’analyse à la probabilité des décisions rendues à la pluralité des voix.* (Edward Elgar Publishing, 1994).

2. Galton, F. Vox Populi. *Nature* **75**, 450–451 (1907).

3. DeGroot, M. H. Reaching a consensus. *J. Am. Stat. Assoc.* **69**, 118–121 (1974).

4. Boland, P. J. Majority systems and the Condorcet jury theorem. *J. R. Stat. Soc. Ser. Stat.* **38**, 181–189 (1989).

5. Kao, A. B. & Couzin, I. D. Modular structure within groups causes information loss but can improve decision accuracy. *Philos. Trans. R. Soc. Lond. B. Biol. Sci.* **374**, 20180378 (2019).

6. Galton, F. The ballot-box. *Nature* **75**, 509–510 (1907).

7. Chandrasekhar, A. G., Kinnan, C. & Larreguy, H. Social networks as contract enforcement: evidence from a lab experiment in the field. *Am. Econ. J. Appl. Econ.* **10**, 43–78 (2018).

8. Golub, B. & Jackson, M. O. Naïve learning in social networks and the wisdom of crowds. *Am. Econ. J. Microecon.* **2**, 112–149 (2010).

9. Clauset, A., Newman, M. E. J. & Moore, C. Finding community structure in very large networks. *Phys. Rev. E* **70**, (2004).

10. Newman, M. E. J., Strogatz, S. H. & Watts, D. J. Random graphs with arbitrary degree distributions and their applications. *Phys. Rev. E* **64**, (2001).

11. Steger, A. & Wormald, N. C. Generating random regular graphs quickly. *Comb. Probab. Comput.* **8**, 377–396 (1999).

12. Barabási, A.-L. & Albert, R. Emergence of scaling in random networks. *Science* **286**, 509–512 (1999).

13. Habermas, Jürgen. *The structural transformation of the public sphere: an inquiry into a category of bourgeois society*. (MIT Press, 1989).

14. Fraser, N. Rethinking the public sphere: A contribution to the critique of actually existing democracy. *Soc. Text* 56 (1990) doi:10.2307/466240.

15. Warner, M. *Publics and counterpublics*. (Zone Books, 2010).
