# Supplementary figures and images for: The functional aspects of selective exposure for collective decision-making under social influence
Source: Sci Rep. 2024 Mar 17;14:6412. doi: 10.1038/s41598-024-56868-8 (PMC10944847; doi:10.1038/s41598-024-56868-8)

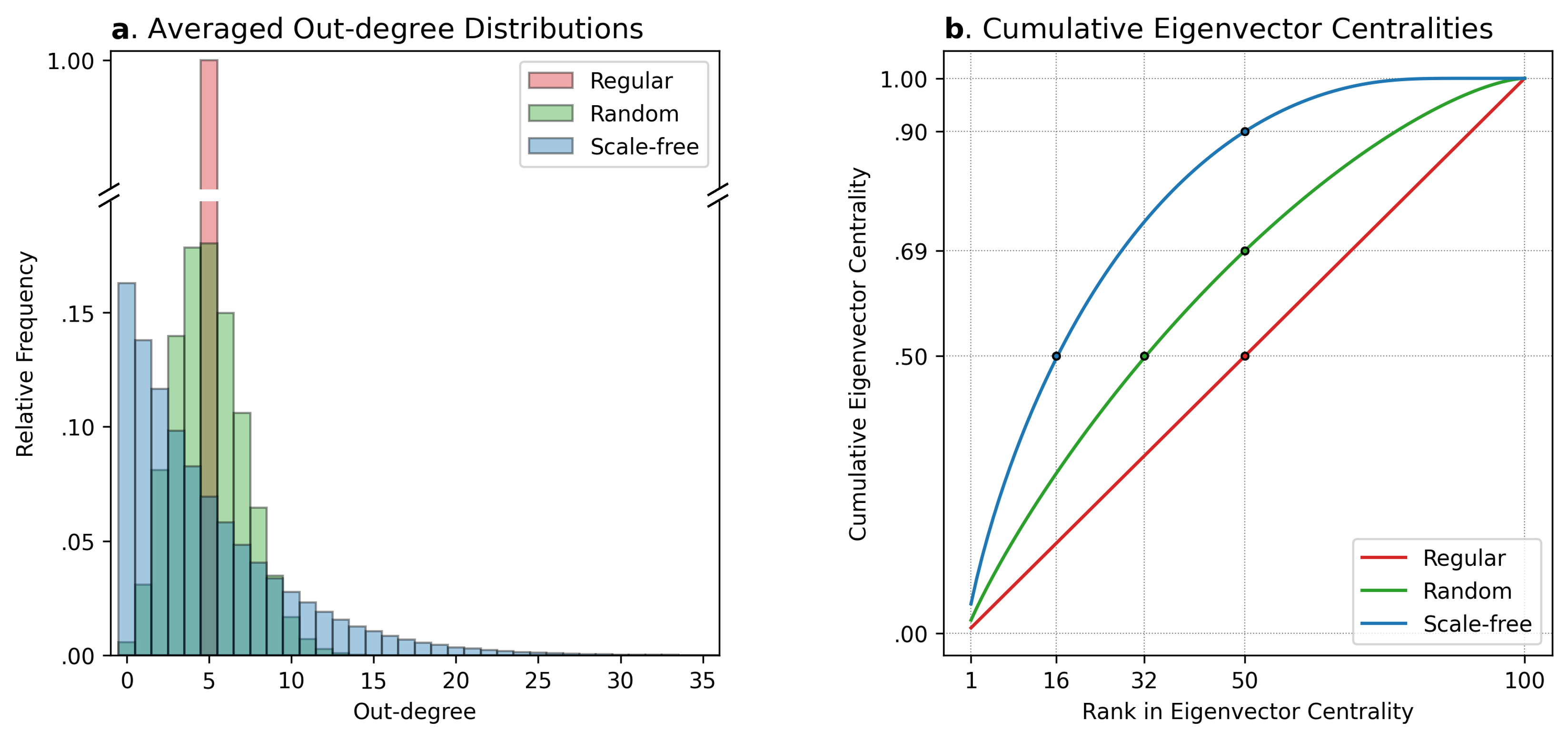

Supplement: Supplementary file 2 — Supplementary Information 2. [file 41598_2024_56868_MOESM2_ESM.png]

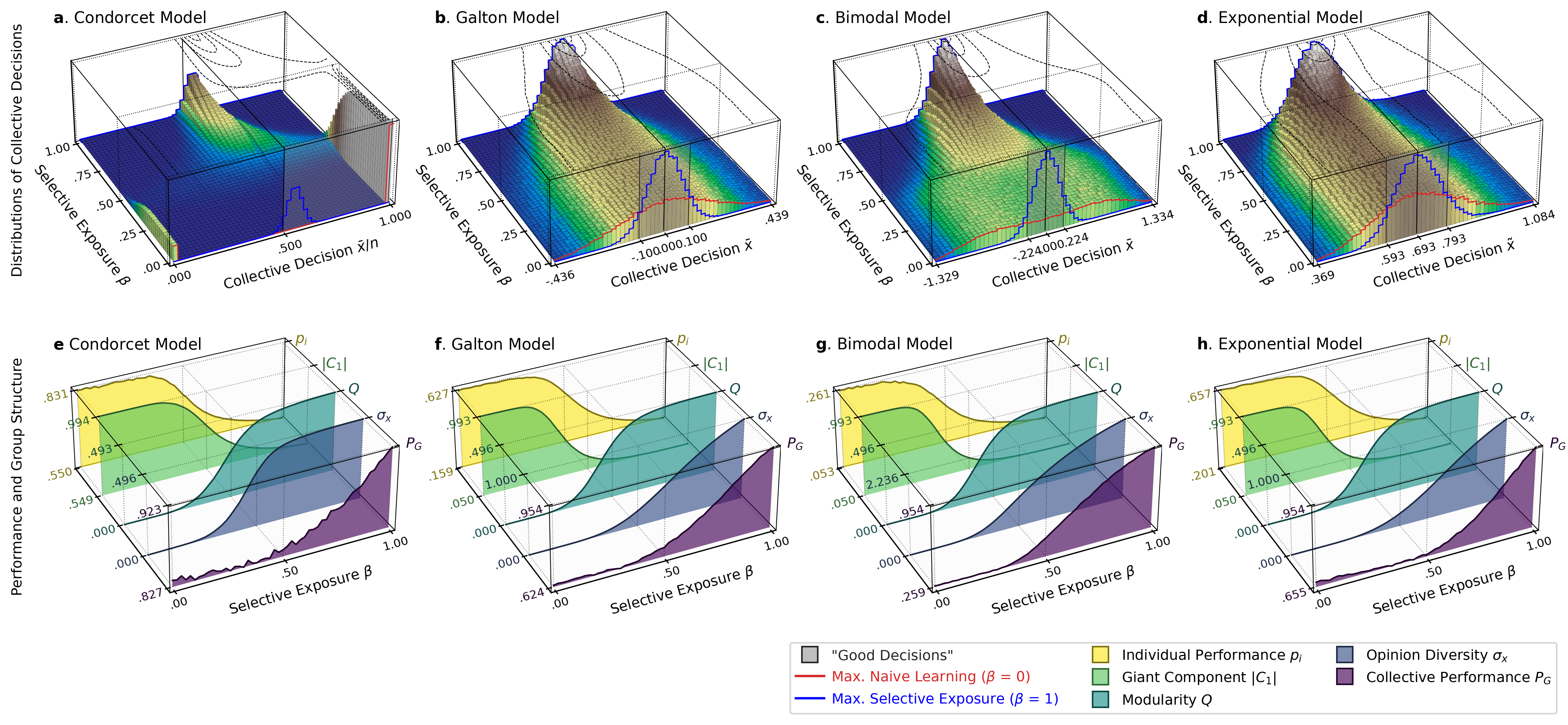

Supplement: Supplementary file 3 — Supplementary Information 3. [file 41598_2024_56868_MOESM3_ESM.png]

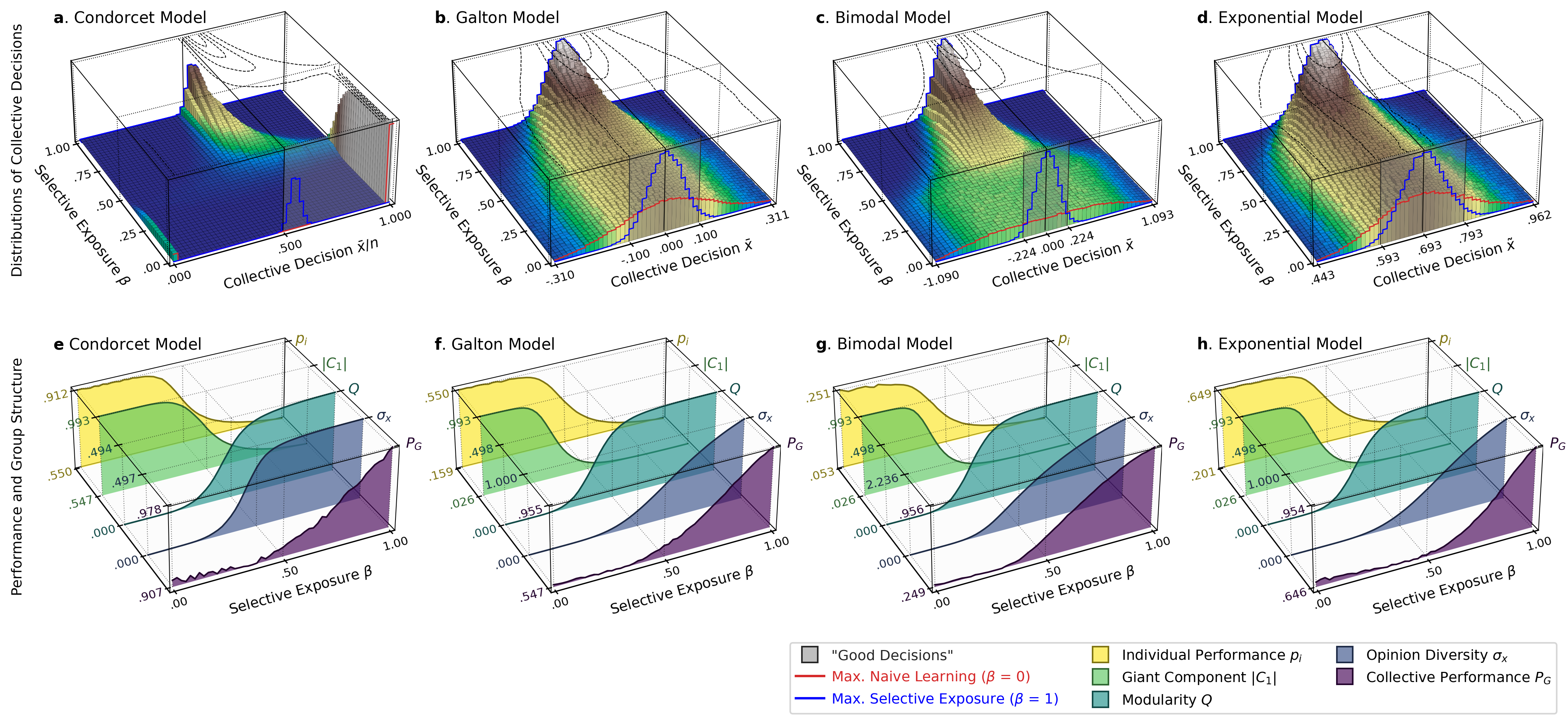

Supplement: Supplementary file 4 — Supplementary Information 4. [file 41598_2024_56868_MOESM4_ESM.png]

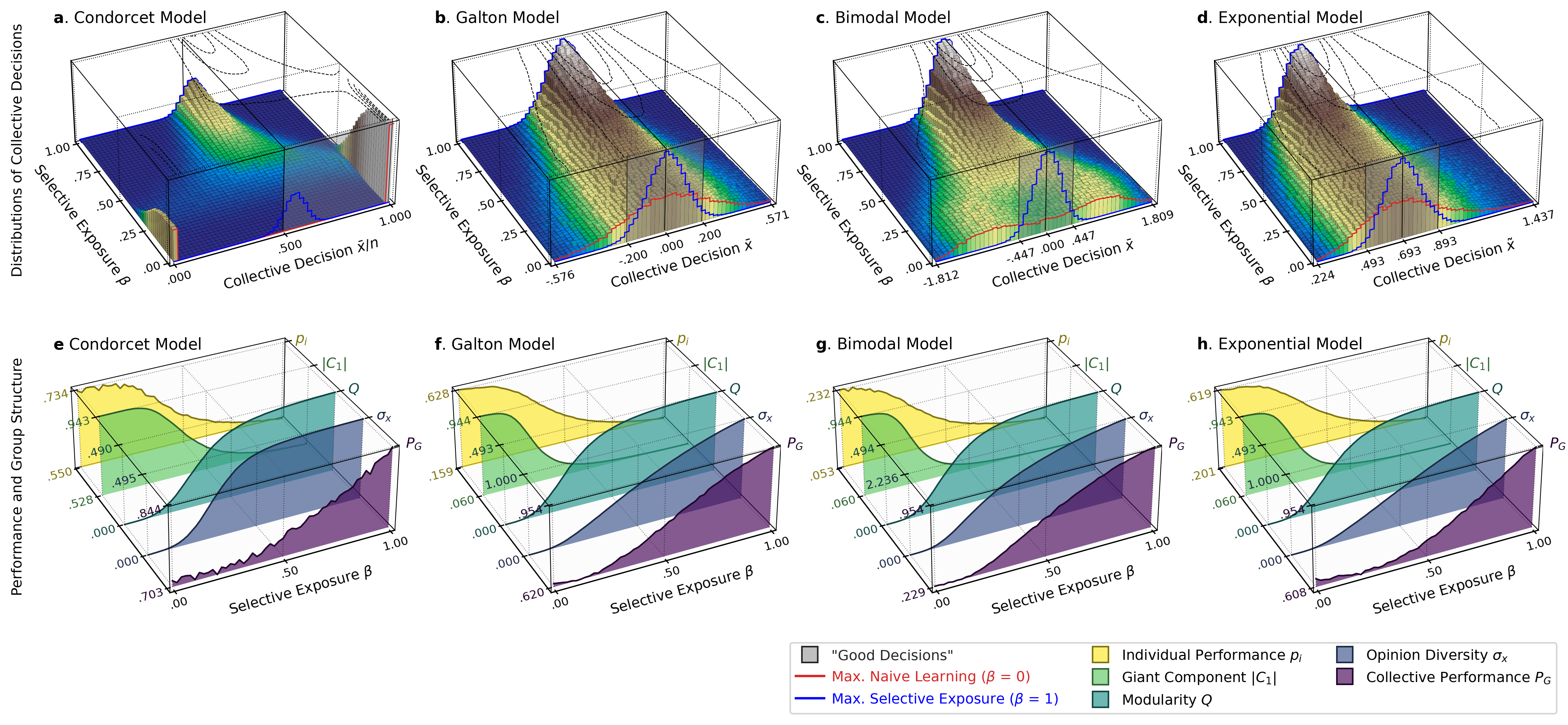

Supplement: Supplementary file 5 — Supplementary Information 5. [file 41598_2024_56868_MOESM5_ESM.png]

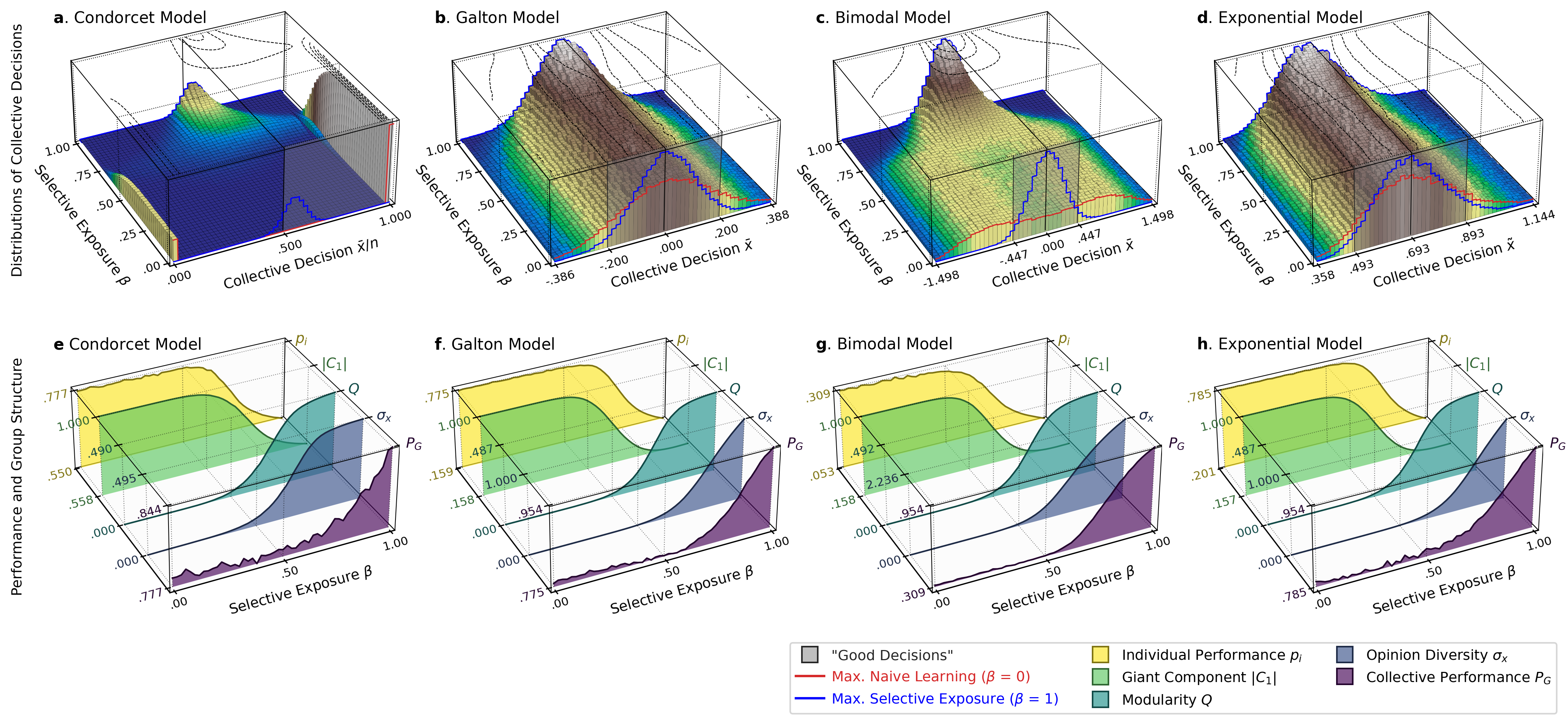

Supplement: Supplementary file 6 — Supplementary Information 6. [file 41598_2024_56868_MOESM6_ESM.png]

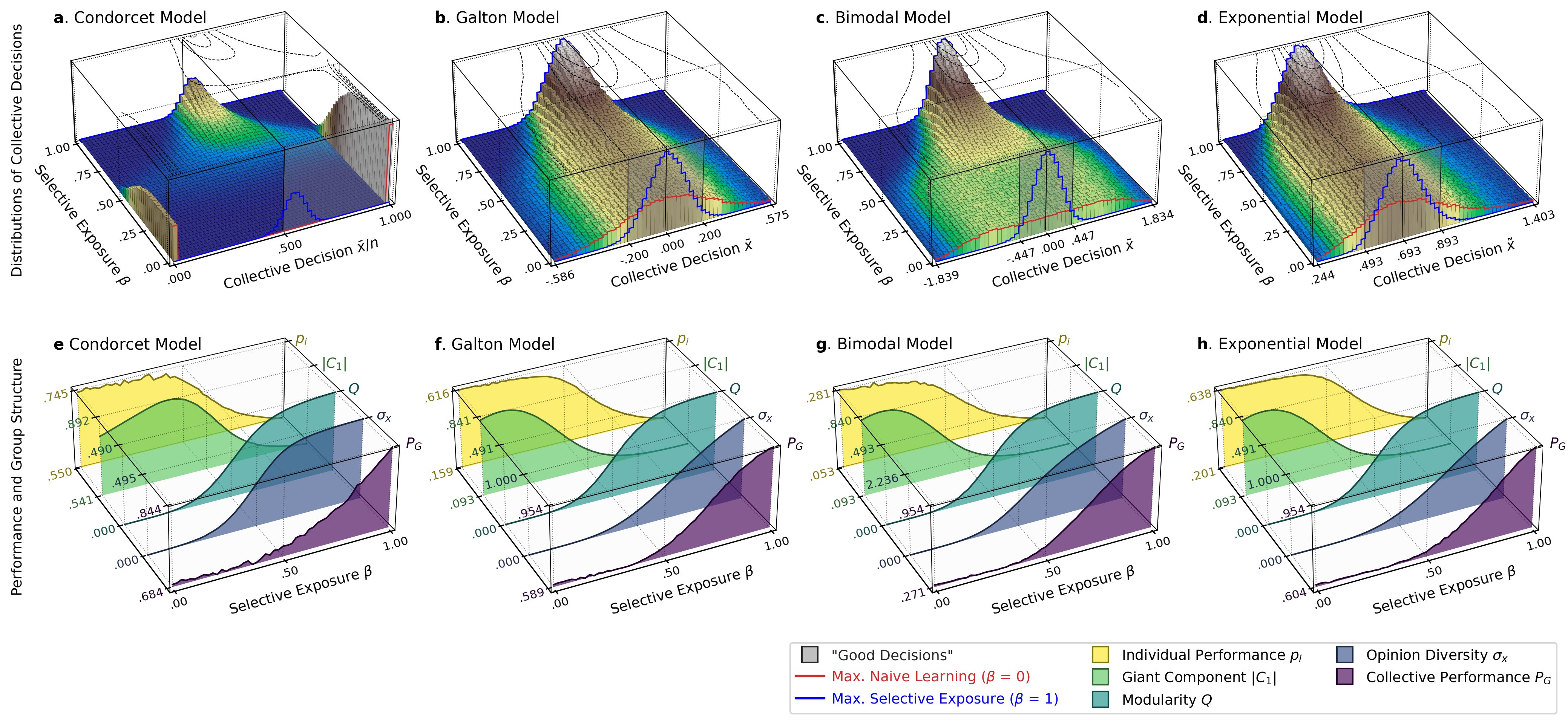

Supplement: Supplementary file 7 — Supplementary Information 7. [file 41598_2024_56868_MOESM7_ESM.png]

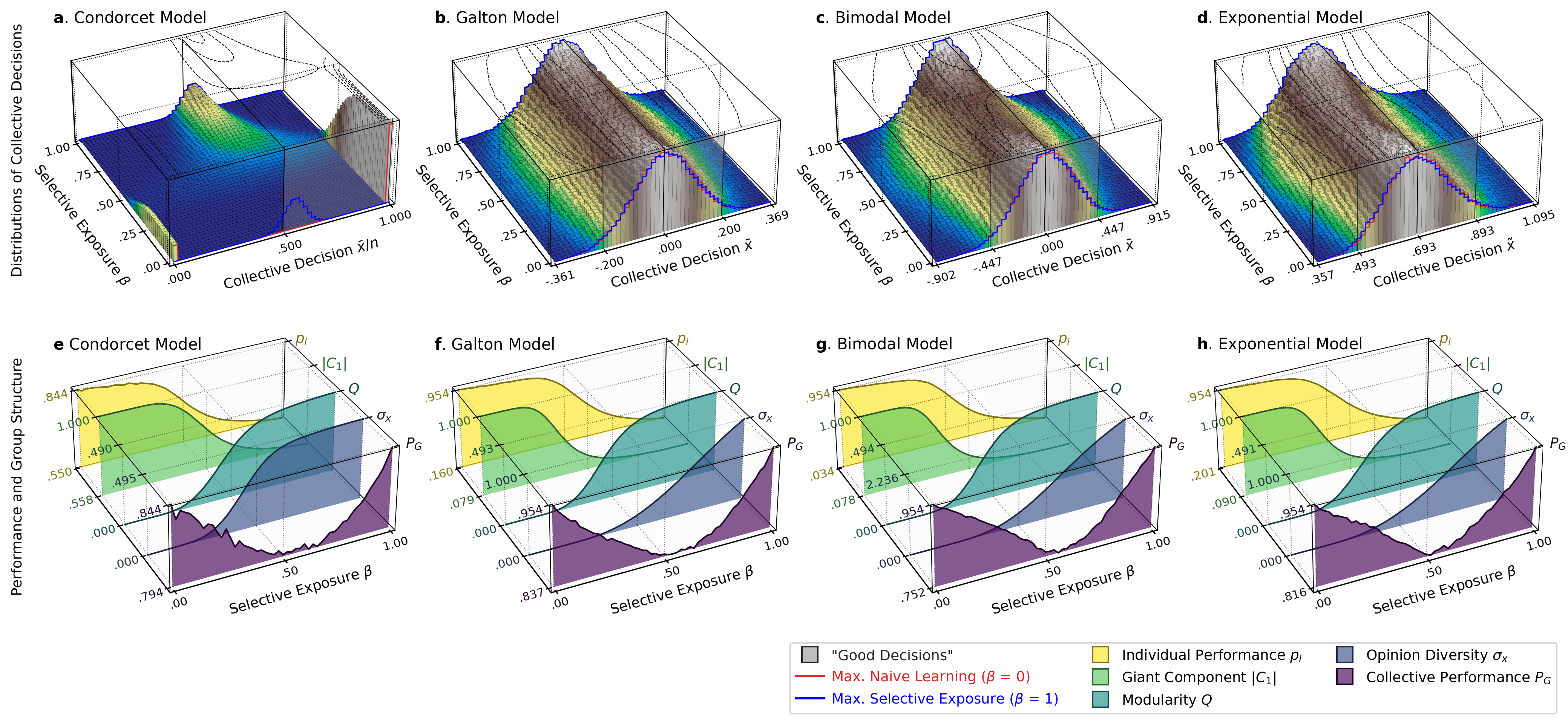

Supplement: Supplementary file 8 — Supplementary Information 8. [file 41598_2024_56868_MOESM8_ESM.png]
